# Recent development of organic–inorganic hybrid photocatalysts for biomass conversion into hydrogen production

**DOI:** 10.1039/d2na00119e

**Published:** 2022-04-19

**Authors:** Ashil Augustin, Chitiphon Chuaicham, Mariyappan Shanmugam, Balakumar Vellaichamy, Saravanan Rajendran, Tuan K. A. Hoang, Keiko Sasaki, Karthikeyan Sekar

**Affiliations:** Sustainable Energy and Environmental Research Laboratory, Department of Chemistry, SRM Institute of Science and Technology Kattankulathur Tamil Nadu 603203 India karthiks13@srmist.edu.in karthik.keyan02@gmail.com; Department of Earth Resources Engineering, Kyushu University Fukuoka 819-0395 Japan; Departamento de Ingeniería Mecánica, Facultad de Ingeniería, Universidad de Tarapacá Avda. General Velásquez 1775 Arica Chile; Institut de Recherche d’Hydro-Québec 1806, boul. Lionel-Boulet Varennes Québec J3X 1S1 Canada

## Abstract

Over the last few years, photocatalysis using solar radiation has been explored extensively to investigate the possibilities of producing fuels. The production and systematic usage of solar fuels can reduce the use of fossil-based fuels, which are currently the primary source for the energy. It is time for us to exploit renewable sources for our energy needs to progress towards a low-carbon society. This can be achieved by utilizing green hydrogen as the future energy source. Solar light-assisted hydrogen evolution through photocatalytic water splitting is one of the most advanced approaches, but it is a non-spontaneous chemical process and restricted by a kinetically demanding oxidation evolution reaction. Sunlight is one of the essential sources for the photoreforming (PR) of biomass waste into solar fuels, or/and lucrative fine chemicals. Hydrogen production through photoreforming of biomass can be considered energy neutral as it requires only low energy to overcome the activation barrier and an alternate method for the water splitting reaction. Towards the perspective of sustainability and zero emission norms, hydrogen production from biomass-derived feedstocks is an affordable and efficient process. Widely used photocatalyst materials, such as metal oxides, sulphides and polymeric semiconductors, still possess challenges in terms of their performance and stability. Recently, a new class of materials has emerged as organic–inorganic hybrid (OIH) photocatalysts, which have the benefits of both components, with peculiar properties and outstanding energy conversion capability. This work examines the most recent progress in the photoreforming of biomass and its derivatives using OIHs as excellent catalysts for hydrogen evolution. The fundamental aspects of the PR mechanism and different methods of hydrogen production from biomass are discussed. Additionally, an interaction between both composite materials at the atomic level has been discussed in detail in the recent literature. Finally, the opportunities and future perspective for the synthesis and development of OIH catalysts are discussed briefly with regards to biomass photo-reforming.

## Abstract 2

In this review, we mainly summarize the important progress in the development of organic–inorganic hybrid photocatalysts for the production of hydrogen using biomass and its derivatives. Recent progresses and challenges are also discussed.

## Graphical abstract

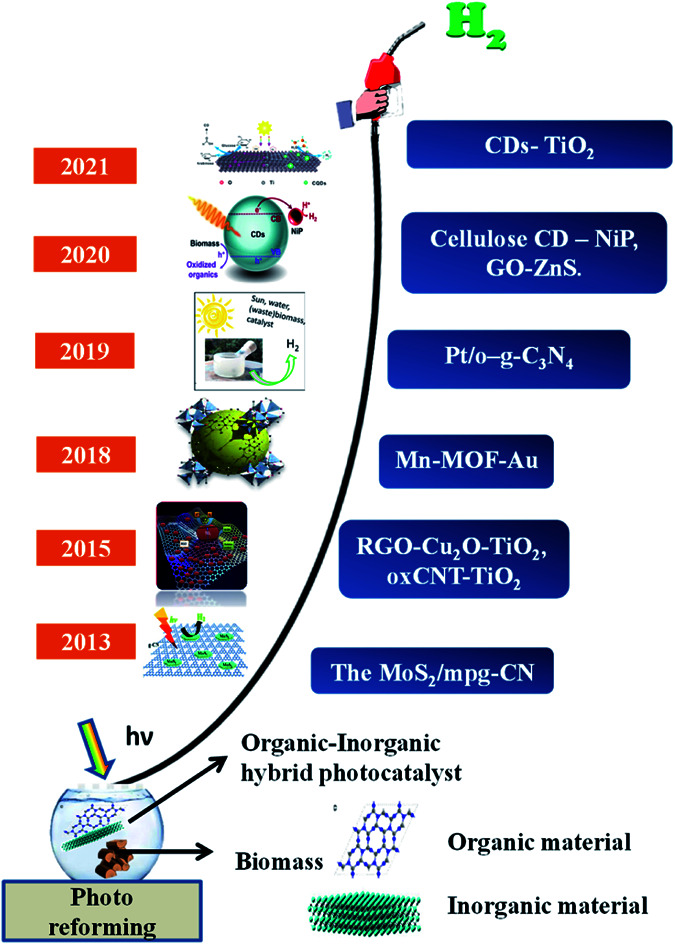

## 1. Introduction

In recent years, there has been a phenomenal improvement in the standard of living for mankind with the advancements in science and technology. Unfortunately, the rising energy demand and a faster product life cycle have led to the depletion of energy sources, along with environmental and waste issues. To solve these issues, it is highly essential to look toward sustainable and efficient energy sources. Photocatalytic hydrogen production has received worldwide attention, as hydrogen has the potential to evolve as a fuel for the post-fossil fuel era. Solar energy conversion into chemical fuel is considered an effective alternative technology for addressing fuel shortages and environmental concerns. Hydrogen has emerged as an adaptable choice for carbon-free transportation, energy source, and other sectors as countries around the world aim for a carbon-free society driven by a sustainable economy.^1^ However, almost all of the hydrogen produced comes from non-renewable fuel sources, like coal, oil and natural gas.^2^ More than 96% of the H_2_ produced globally is from the steam reforming of fossil fuels. Furthermore, less than 2% of the annual hydrogen production is from electrolysis using renewable electricity.^3^ Non-renewable resources cannot be considered feasible sources as they cannot be restored within the timescale, and their carbon dioxide emission contributes to global warming. Sustainable hydrogen generation from renewable sources can be a promising and eco-friendly way to address global energy problems and minimize the reliance on fossil fuels. This can only take effect by changing our strategy in resource generation, usage, and disposal.^4^ Currently, society follows the fossil-based methods that could offer financial benefits. However, in the long run, this will be a challenge for us to conserve the limited resources and reduce the emission of hazardous gases. To move towards a carbon-free society, there should be a slow and steady turnaround in the production and utilization of energy and materials. The energy domain, which includes transportation, power and industry, is responsible for more than 75% of greenhouse gas emissions.^5^ To reduce the emission of carbon into the atmosphere, a few methods are used, such as using renewable energy sources instead of fossil fuels, reducing the use of electricity and fossil fuels in industries and public sectors, and capturing CO_2_ as a derivative of fossil fuel combustion before entering the atmosphere. However, these methods have their own limitations and they cannot be considered as a long-term remedy towards a green economy. Renewable energy sources, such as solar, wind, and hydropower, are widely used for energy production, despite their low conversion efficiency. In this aspect, green hydrogen can be considered as an energy carrier for the future. To produce hydrogen, solar energy should be considered because it is the ultimate source of renewable energy.^6^ More than 48% of the sunlight spectrum consists of UV-visible radiation.^7^ So, the most effective and promising approach for resolving energy and ecological challenges is the successful conversion of visible sunlight into chemical energy stored in hydrogen, which has recently captured a lot of attention.^8^ Green hydrogen is a clean, flexible energy source that supports the zero carbon emission norms. H_2_ contains an approximate energy of 122 kJ g^−1^, which is considerably higher than gasoline (47 kJ g^−1^), coal (15 kJ g^−1^), natural gas (40 kJ g^−1^) or any other fossil-based fuels.^9^ Hydrogen is light, flammable, and storable. It has high-energy content and can be used for various purposes, such as carbon-free fuel production, production of fertilizers, and metallurgy.^10^ Therefore, it is necessary to produce green hydrogen from never-ending sources to look towards a clean, sustainable society.

One of the most advanced approaches for hydrogen evolution is the photocatalytic water splitting using solar energy. The reduction and oxidation of H_2_O to H_2_ and O_2_ occur simultaneously in this process.^11^ The burning of the hydrogen fuel produced using solar radiation does not lead to the emission of CO_2_ gases, as the carbon cycled is closed. The photocatalytic water splitting (WS) is being developed as a sustainable technology that can bring a change to offset the 830 Mt of annual CO_2_ emission due to the steam reform of fossil fuels by producing clean hydrogen.^12^ Many large-scale photocatalytic hydrogen production processes with decent yields have been developed recently,^13–16^ which include the NiSCd_*x*_Zn_1−*x*_S catalyst and Na_2_SO_3_ with scavenger yields of 10 400 μmol m^−2^ h^−1^.^17^ Another notable achievement was attained by the Cu/TiO_2_ photocatalyst with glycerol producing 1240 μmol L^−1^ of hydrogen.^18^ The photooxidation process can be avoided by using a hole scavenger (sacrificial agent), which scavenges the holes before it undergoes oxidation. They are typical scavenging agents, like ethyl alcohol, methanol, sulfides or thiosulfates. For an effective functioning of the scavenger, the redox potential should be above the oxidation level of the catalyst, which facilitates the smooth transfer of the hole to the sacrificial agents.^19^ Even though it is widely encouraged, the large-scale production of hydrogen still faces a few issues, such as reusability of the photocatalyst, the use of expensive metals as sacrificial agents, the separation of the O_2_ and H_2_ mixture, and the inefficient use of visible light. WS is a non-spontaneous chemical process [change in Gibbs free energy, (Δ*G*^0^) = +237 kJ mol^−1^ at 25 °C], and it is restricted by the kinetically and energetically demanding oxidation evolution reaction.^20^ The difference in energy is responsible for the maximum limit of wavelength of the photons irradiated, which is responsible for the initiation of the reaction. The energy can be calculated using the equation Δ*E*^0^ = Δ*G*^0^/*nF*, where *n* represents the number of exchanged electrons and *F* is the Faraday constant. In the case of the water splitting reaction, ‘*n*’ has a value of 2, and Δ*E*^0^ is calculated as 1.23 V. This depicts that the photon should possess a minimum energy of 1.23 eV with a wavelength shorter than 1008 nm (*λ* = *hc*/Δ*E*).^21^ Those reactions having positive Gibbs free energy values normally have high activation energy, *i.e.*, the thermodynamic barrier of WS is −1.23 V, so the electrons require high energy to carry out the chemical reaction. The overpotential is necessary to overcome the kinetic restrictions, which significantly increase especially on the half-oxidation. The larger overpotential showcases the wider band gap, which depicts a higher charge transfer, also leading to the absorption of shorter wavelength radiation.^22^ Most of the photocatalysts are absorbed in the UV region but limited UV portion (3–5%) in the solar spectrum, resulting poor photocatalytic activity. To achieve a higher productivity for photocatalytic reactions, it is critical to use visible radiation (44 percent), which accounts for the majority of sunlight radiation.^23^ So, only a few visible light absorbing photocatalysts are used for the WS reaction.^24^

## 2. Scientific analysis of photoreforming

**Fig. 1:**
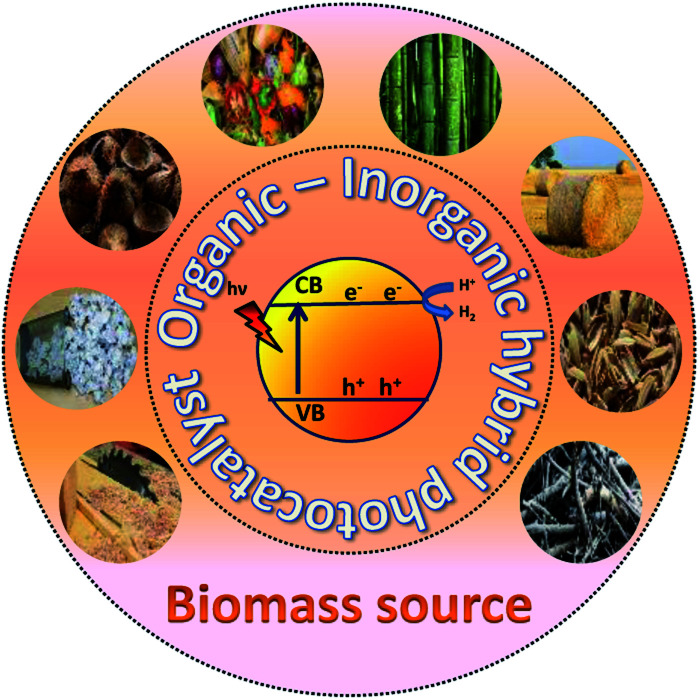
Plausible schematic diagram for photocatalytic hydrogen production from various biomass sources.

To bring in a solution, photocatalytic reactions with Δ*G*^0^ < 0 are studied. These total reactions involve hydrogen production on one electrode, while hole scavengers such as triethanolamine, methanol, and lactic acid are used. They work on the other electrode, and offer a faster rate than the oxygen evolution reaction.^25^ Photoreforming (PR) is an alternative method for hydrogen production from water and organic substrates, which has the potential to overcome the outstanding constraints.^26^ In PR, the photocatalyst absorbs radiation from the solar spectrum. Electrons in the lower energy level (valence band) are then excited to the highest energy level (conduction band). Consequently, an electron and a hole are evolved. Most of these pairs will recombine without any further reaction. If not, these holes and electrons are carried to the surface of the photocatalytic material. Strategies, such as heterojunction design, construction of intramolecular donor acceptor system, spin polarization regulation and excitation dissociation regulation, are used to inhibit the charge transfer recombination in photocatalysts. The formation of a heterojunction facilitates an efficient charge separation and charge carrier transfer through the interface. The D-A system allows for high speed charge transfer within the photocatalyst. The free electrons increase by regulating the dissociation of the electron–hole pair into free carriers. The charge recombination decreased and the efficiency of the electron transfer increased *via* controlling the electron spin polarization. Even though these strategies exhibit the ability to inhibit the recombination of excitons, these strategies have their own limitations. The incorporation of electron donors or electron acceptors with organic linkers may reduce the length of the conjugated system, making it difficult for the migration of the electron between the donor and acceptor. Defects like a lattice mismatch can exist between the two semiconductors that make up the heterojunction photocatalyst, and the defects become the recombination center of the photogenerated carriers. The exciton dissociation and electron spin polarization regulation have not been explored much, and the mechanism needs to be further studied.^27^ The electrons and holes reduce water to hydrogen and oxidize organic substrates into small molecules, respectively.^28^ These substrates can be plastic waste, food materials, and biomass. This can effectively aid in avoiding the use of costly sacrificial agents, while offering effective conversion of waste materials.^29^ PR can be implemented at room temperature with solar energy as the external energy source to produce clean hydrogen fuel. PR brings forward a different approach of both introducing a method for waste disposal and producing useful organic materials. The thermodynamic energy barrier of the PR mechanism for most of the organic substrates is energetically neutral. For example, the photoreforming of ethylene glycol needs Δ*G*° of +9.2 kJ mol^−1^ (standard electrode potential − 0.01 V) at room temperature.^28^ PR prefers substrates having minimal complexity, high polarity/hydrophilicity, water solubility, and functional groups that adsorb to the surface of the photocatalyst, according to studies with simple molecules (Fig. 1).^30^

The photoexcited condition of the semiconductor is used for the photo-reforming of the biomass at ambient temperature and pressure. The photocatalyst will absorb the light radiation with energy higher than its band gap, through which the valence band electrons jump into the conduction band upon absorbing the proper energy. Electrons in the conduction band have reducing property, so it can facilitate the hydrogen formation (HER, mentioned in eqn (1)). The hole in the valence band, on the other hand, can accelerate the biomass oxidation reaction (BOR). Eqn (2) represents the BOR for glucose. The hydrogen evolution through water splitting is challenging, as it has a high thermodynamic barrier and produces H_2_ and O_2_ mixture (eqn (3) and (4)). Eqn (5) gives the overall reaction involved in the PR of the biomass, which is energy-neutral. Thus, it requires only low energy to overcome the activation barrier. So, the PR of the biomass can be carried out using photons with lower energy levels, which are largely available in the solar spectrum.^23^

1 2H^+^ + 2e^−^ ⇄ H_2_, *E*^0^ = 0 V *vs.* RHE

2 C_6_H_12_O_6_ + 6H_2_O ⇄ 6CO_2_ + 24H^+^ + 24e^−^, *E*^0^ = −0.001 V *vs.* RHE

3
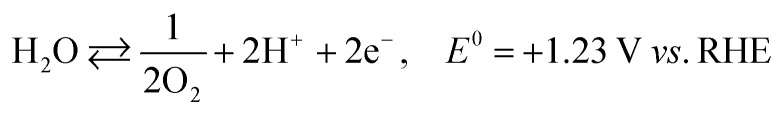
4
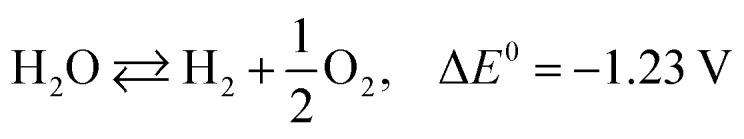

5 C_6_H_12_O_6_ + 6H_2_O ⇄ 12H_2_ + 6CO_2_, Δ*E*^0^ = +0.001 V

A desirable substrate for PR should contain as many of these characteristics as feasible, and also be generated from non-recyclable waste materials. H_2_ is currently in the growing stage and because it has a low market value, alternative PR products are sought after. Furthermore, the specific photocatalytic processing of renewable feedstocks into lucrative organic valuables is a seriously debated topic.^31^

### 2.1 Photo-reforming feedstock

On a global basis, there is increasing attention in developing waste-to-energy resolutions for municipal solid waste (MSW), fueled by ecological concerns regarding inadequate disposal techniques.^32^ Waste produced by industries largely exceeds those produced at the municipal level. However, due to the shortage in the availability of global statistics on the production and disposal of industrial wastes, we will concentrate mainly on the MSW statistics. Every year, about 2 billion tonnes of MSW are produced around the world. By 2050, global garbage production is expected to increase by 70%, which is estimated to 3.4 billion tonnes. More than 70% of MSW is sent to landfill sites or disposed of by open dumping.^33^ Recycling accounts for 13% of the world's municipal solid trash. Every year, 93.9 million tonnes of MSW are recycled or composted.^34^ The absence of an appropriate waste management has a significant impact on public health and society. It is estimated that implementing trash prevention, recovery and recycling measures may cut global carbon emissions by 20 percent.^35^ Food waste and biomass (46%), paper products (17%), and plastic (12%) make up the majority of MSW globally, with textile, metal, leather, and other trash accounting for the remaining 25%. There is 75% of MSW components that contain organic substrates and can be potentially used for the PR. This review will focus on producing clean hydrogen and organic substrates from waste materials, and thereby evaluate the possibility of fuel production and waste management simultaneously.

The most suitable waste component available for PR is biomass, which includes sucrose, glucose starch and wood.^36^ Around 4 Gt of biomass waste is annually produced from various sources, like farming and industry residues. The biomass-derived substrates like carbohydrates and alcohols can be used to produce hydrogen from sewage food, beverages, and paper industry waste materials. Lignocellulose, a combination of cellulose, hemicellulose, and lignin, is the main component of biomass.^23^ Hardwoods have more cellulose and hemicellulose content (78%) compared to softwoods (70%), whereas softwoods have higher lignin content (29.2%) than hardwoods (21.7%).^37^ Cellulose and hemicellulose are long chain carbohydrates (C_6_H_12_O_6_ or C_5_H_10_O_5_) that can undergo PR because of the polarity and hydroxyl functional group present.^38^ The hydrophobic nature of lignin makes it difficult for photo-reforming. Around 55–95 wt% of the chemical content can be reformed from various biomass sources, depending upon the photocatalyst used.^39^

The practicality of biomass-derived substrates has been carried out using different carbohydrates, organic acids, and other derivatives. The amount and type of the substrate, the reaction media, and temperature all influence the yield of hydrogen. The position of lower and higher energy bands of the photocatalyst has a major role in the selectivity of the product. Band gap engineering is widely carried out to obtain the desired product as chemicals and reaction pathways, as the oxidation half is controlled by the holes of the photocatalyst.^40^ X-ray photon spectroscopy is used to obtain the band gap of the photocatalyst used.^41^ Various methods have been carried out for band gap engineering, such as vacancy creation, quantum dots, heterojunctions, and solid solutions. This allows for the exploitation of the band gap of the catalyst to improve its activity and efficiency. Cocatalyst loading is also an effective method to improve the photo-oxidation. The loadings of cocatalyst such as (Pt, Au, Ag) boost the electron transportation and charge separation. By boosting the photoinduced electron and hole separation, the inserted metal species can speed up the reaction process.^20^ These metals contain active sites for the electrons, which are photogenerated, thereby inducing a charge separation. To improve the biomass functionality of the catalyst, procedures like enzymatic exfoliation, acid/base addition, and increased ionic liquid solubilization have been utilized with photocatalysis.^42,43^ The initial step in converting biomass to hydrogen is to use TiO_2_, with Pt and RuO_2_ serving as the reduction and oxidation co-catalysts, respectively. The starch and cellulose yields 320 μmol/20 h and 244 μmol/20 h of hydrogen, respectively, using the RuO_2_/TiO_2_/Pt photocatalyst.^44^ The photocatalytic fuel production has been carried from this pioneer work, but most of the studies are based on TiO_2_ materials. Even though TiO_2_ is responsive and of low cost, its large band (3.2 eV) hinders it from absorbing solar radiation to only UV light. CdS has also been considered as the conventional photocatalyst in PR of biomass, but its toxic nature and vulnerability to corrosion is considered as drawbacks.^45,46^

### 2.2 Methods of hydrogen production from biomass

**Fig. 2:**
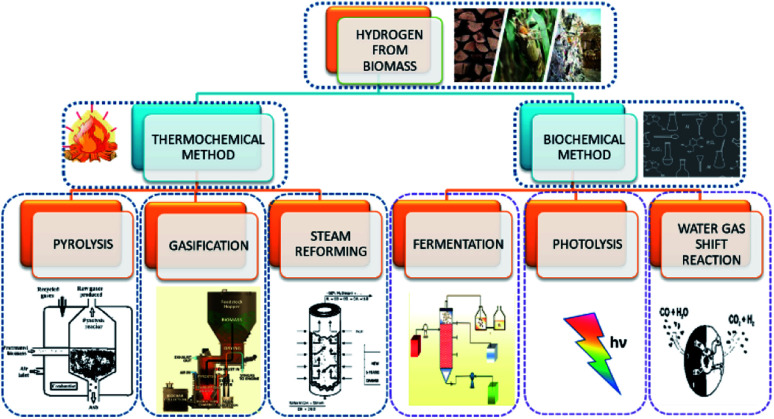
Different biomass reforming processes for hydrogen production.

Hydrogen evolution through biomass can be classified as thermochemical and biological processes. The former can be described as the four processes of (i) combustion, (ii) pyrolysis, (iii) liquefaction, and (iv) gasification (Fig. 2). The thermochemical process involves the decomposition of biomass thermally, with or without the aid of a catalyst. The product obtained in thermal decomposition such as biomass gasification is usually carried out with the help of external heat (combustion). It is controlled by major parameters, such as the temperature, rate of heat, time for reaction and used catalyst. The process of biomass gasification includes the partial oxidation of the substrate that produces gaseous products.

The formation of tar and ash formation during the process is the major disadvantage of gasification. With the use of some additives such as dolomites, char can be used to resolve the former problem, and fractionation and leaching can be used to avoid the formation of ash in the reactor. Its advantages include low-cost production and better yield. Coal gasification is widely and efficiently used, but it is highly dependent on hazardous fossil fuels. The high installation and maintenance cost is also a drawback for coal gasification. Steam methane reforming is also a relatively stable and efficient form of hydrogen production, but the production of CO_2_ and high operating temperature are the main challenges.

On the other hand, biological processes are mainly classified as (i) direct and indirect bio-photolysis, (ii) hybrid system, (iii) dark fermentation, and (iv) photo-fermentation, as they are eco-friendlier and less energy-consuming. Hydrogen-producing microbes, like nitrogenase and hydrogenase, carry out these operations. Direct bio-photolysis of producing hydrogen is a process that converts sunlight into chemical energy stored in hydrogen, using microalgae photosynthetic systems. Even though the consumption of the substrate is high, the efficiency is very low. Indirect bio-photolysis involves the conversion of solar light by multiple steps mechanism and the yield obtained is comparatively higher than the direct bio-photolysis. Fermentation can be carried out to produce hydrogen in the presence of light and dark conditions by bacteria at 30–80 °C. Methane fermentation under aerobic condition is one of the major methods, but the yield of hydrogen is low. The fermentation is kinetically slow, but the disposal of fermentation waste is an issue. Even though the biological conversion requires low energy input, it is only carried out on a small scale and produces by-products.

## 3. Photocatalyst materials

**Fig. 3:**
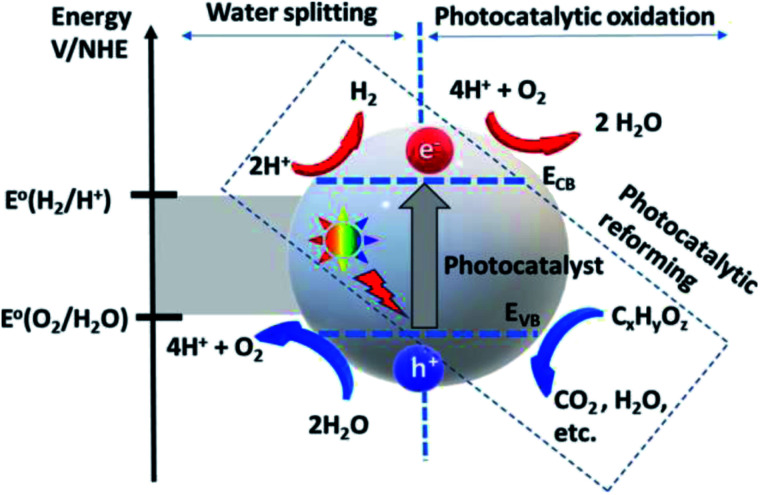
Photocatalytic water splitting and oxidation mechanism.^57^ Copyright 2020, International Journal of Hydrogen Energy.

Scientists have been using different photocatalytic materials for hydrogen production from biomass and its derivatives to find a new path for fuel generation other than the splitting of water. In 1980, the first reported hydrogen production from carbohydrates was published using the RuO_2_/TiO_2_/Pt photocatalyst and 500 W xenon light.^44^ Hydrogen is also produced from other biomass sources, like starch, dead flies, cellulose, algae and waste materials, using similar methods.^47,48^ Researchers have encouraged the use of doped TiO_2_ (metal and non-metal doped TiO_2_) (simple and mixed oxides), sulphides (cadmium, zinc), carbon nanomaterials, covalent-organic frameworks (COFs), metal–organic frameworks (MOFs), hybrid inorganic–organic mesoporous materials, polymeric semiconductors, and co-catalysts (metal/metal oxide) for solar absorption. This shows the wide possibility of biomass conversion to hydrogen. Widespread research is underway to develop fine band gap materials and metal-free materials for H_2_ generation *via* photo-reforming, with the goal of enhancing visible light photocatalytic performance, while also improving selectivity toward important compounds and H_2_ evolution (Fig. 3). Extensive work has been carried out with titania-based materials due to their high stability, non-toxicity, abundance, and efficiency.^49,50^ In order to compensate the large band gap and the inability to absorb the light from the visible region, TiO_2_ could be loaded with a cocatalyst (Pt, Pd, Au) and visible light sensitizers (CdS, C_3_N_4_) to enhance the photocatalytic efficiency for hydrogen production.^50,51^ Doping with different transition metals can increase the catalytic efficiency mainly by the reduction of the electron–hole pairs recombination and the enrichment of surface active sites.^52^ As an example, the photocatalytic conversion of cellulose has been carried out and large yield could be obtained from cellulose-immobilized TiO_2_/Pt at 195 μmol.^53^ Photocatalysis and acid hydrolysis of cellulose biomass into hydrogen has been carried out in sulphuric acid medium using Pt/TiO_2_ as the catalyst. The reaction yielded 123 μmol of hydrogen with 0.6 M H_2_SO_4_ concentration and a production of 1320 μmol h^−1^ g_cat_^−1^ was obtained using raw biomass paper pulp in the same condition.^54^ Pd-loaded TiO_2_ has also been studied in the photo-reforming of biomass. The surface-bound cocatalyst can enhance the photocatalytic reaction by binding electrons to its surface and facilitating the hydrogen evolution reaction.^55,56^

**Fig. 4:**
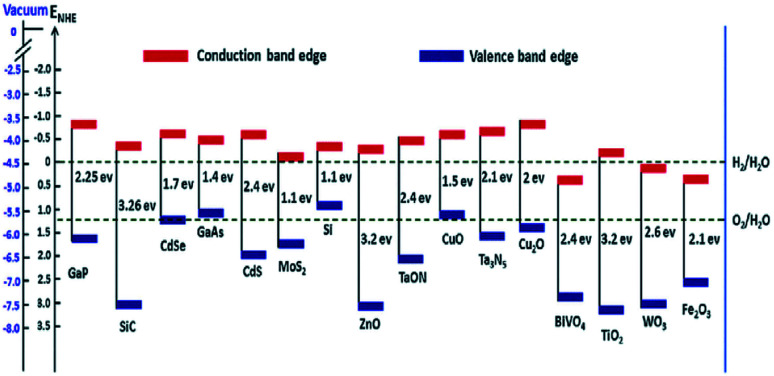
Band edge positions and corresponding redox potential of semiconductors at pH = 0 with respect to the vacuum level and NHE.^60^ Copyright 2016, Nanoscale Horizons.

The deposition of Au metal on the photocatalyst surface has also been effectively used for hydrogen evolution from biomass.^58,59^ The positions of the valence band and the conduction band of the material have a vital role in the HER and OER because these define the suitable energy of absorbed light radiation (Fig. 4).

The photo-reforming of ethanol has been reported using Au/TiO_2_, where the gold particles are supported on the photocatalyst by deposition precipitation method. Under UV irradiation, H_2_ generation yields are extremely high (30 mmol g^−1^ h^−1^) and the yield rises with increasing gold loading.^61^ Once the radiation fall on the photocatalyst surface, the reduction of Au^3+^ to Au (0) is carried out by the electron. This leads to the alignment of the Fermi levels of both TiO_2_ and Au, which is responsible for the quenching of the electron–hole recombination. Another study showed that when using high-pressure Hg lamp of 125 W, the photocatalytic reforming of glucose using the Au/TiO_2_ photocatalyst yielded 1.37 mmol h^−1^ of hydrogen.^62^ A report showed that the photo-reforming of methanol had been carried out using different metal cocatalysts on TiO_2_.^63^ Here, Pt achieves the highest activity and the specific activity order is given by Pt > Au > Pd > Rh > Ag > Ru. This can be explained due to the higher overpotential for the production of H_2_ on TiO_2_, and also due to the work function of noble metals.^64^ The former is the reason for the very low inactivity of TiO_2_, and the latter will lead to the construction of a strong Schottky barrier formed at catalysts interface junction.^65^ The work function of platinum metal is higher, and thus produces more yield in hydrogen evolution compared to other metals. The Schottky barrier is responsible for the decrease in the creation of the electron–hole pairs, and shows better photocatalytic activity.^66^ The photocatalytic reaction's efficiency is still quite low and challenging for practical implementation due to the high recombination rate of the electron–hole pairs and the photocatalyst's inadequate active sites, both of which lead to the low quantum yield and photocatalytic efficiency.^67^

There are alternate methods in which nickel sulphides and oxides are used to avoid the use of costly noble metals for the photo-reforming of biomass material to hydrogen. It has been reported that when TiO_2_ is modified with nickel sulphide and sulphate, the hydrogen yield is 181 μmol from cellulose. The sulphate particles act as a solid acid catalyst, which facilitates the hydrolysis of cellulose. This will lead to the availability of soluble glucose to the catalyst, which quenches the holes and donates electrons. The nickel sulphide acts as a cocatalyst and traps electrons for the reaction.^68^ When nickel oxide is used, a graphitic overlayer is added at the interface. This helps improve the conversion efficiency successfully. This carbon layer is responsible for the weakening of the O–H bonds of the substrates used. The hydrogen produced is reported as 270 μmol g_cat_^−1^ h^−1^ at low temperature and 4150 μmol g_cat_^−1^ h^−1^ at high temperature.^69^ Cadmium sulphide is another commonly used substance, which is recognized for its capability for absorbing visible light due to its high molar extinction coefficient in the visible area. The overall photocatalytic efficiency can also be increased by modification techniques, such as surface plasmon resonance. This enhances photostability, creates new sites on the surface, and suppresses electron–hole recombination.^70^ The activity can further be enhanced by loading the material with cocatalyst metals.

CdS nanosheets loaded with the nickel co-catalyst will reduce the charge recombination, leading to the production of H_2_ and value-added compounds from biomass. For the Au/CdS nanorod catalyst, a yield of 90 μmol h^−1^ g_cat_^−1^ hydrogen from glucose is obtained using visible radiation, which confirms that the self-reducing ability of Au^3+^ to Au increases the separation and transportation of photogenerated excitons.^71^ Another report shows that the lignocellulosic biomass is used to evolve hydrogen by using a stable CdS/SiC composite material at high temperature. The yield obtained is 321 μmol h^−1^ g_cat_^−1^ of hydrogen when Pt is used as the cocatalyst for the reaction using α cellulose as the substrate.^72^ The valorization of cellulose is carried out using CdS/CdO_*x*_ quantum dots, and provides high yield of hydrogen. It is noted that the reaction is implemented without the use of any cocatalyst material or CdS photo-corrosion in the basic medium.^73^

**Fig. 5:**
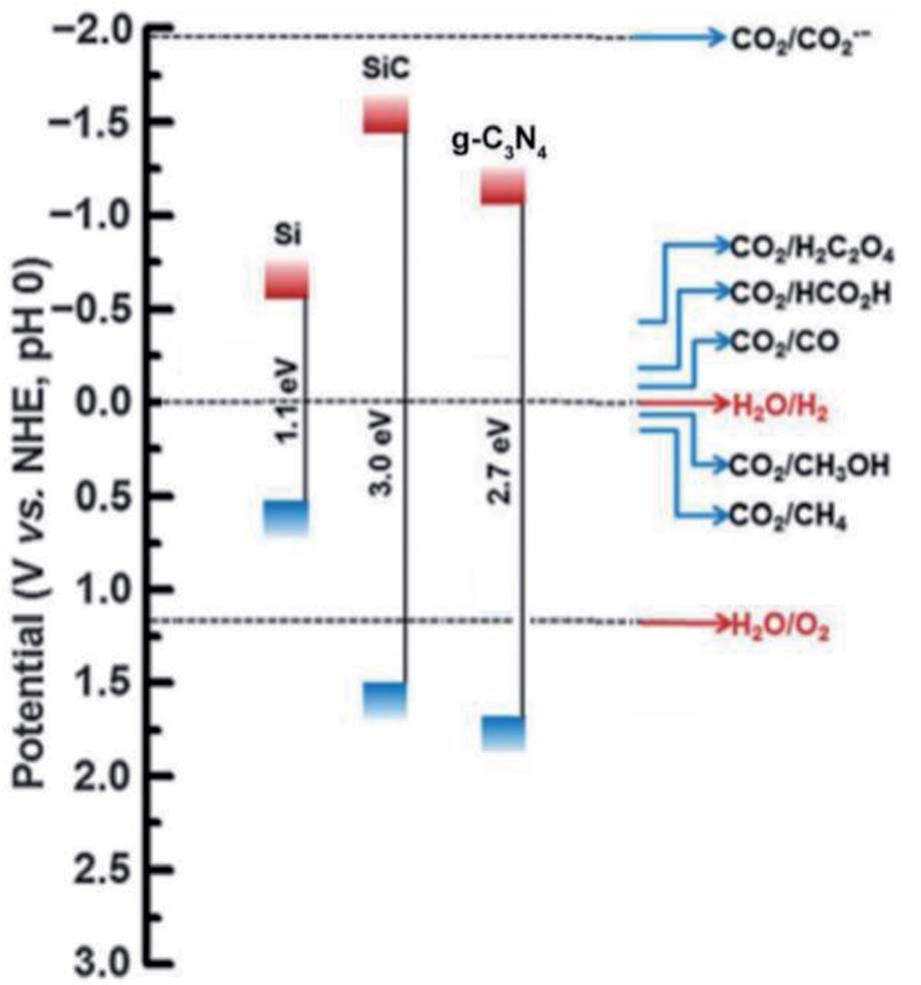
The band gap position of a few metal-free photocatalyst is depicted in the figure.^74^ Copyright 2017, Green Chemistry.

In order to achieve a more sustainable and eco-friendly means to produce H_2_ from biomass sources, the use of metal-free photocatalyst is used potentially. These photocatalysts are basically derived from elements like chalcogens and carbon-based derivatives. This includes graphitic carbon nitride, boron carbides, graphene oxides, carbon quantum dots, and other organic compound photocatalysts that have exhibited promising applications in energy conversion (Fig. 5).^74^ These materials are known for their unique characteristics, such as good thermal and electrical conductivity, stability, bonding effects to produce active sites and tunable structures. The most widely used metal-free photocatalyst is g-C_3_N_4_ towards hydrogen production using visible light. g-C_3_N_4_ is known for its versatile morphology and stability, but its low surface and rate of charge recombination is considered as demerits.^75^ Photo-reforming of lignocellulose using cyanamide-functionalized carbon nitride showed good H_2_ production in the presence of visible light. A maximum yield of 2.62 μmol was obtained under alkaline medium. The same reaction was carried out using saw dust, and a yield of 202 μmol h^−1^ g_cat_^−1^ of hydrogen was obtained. The activity was mainly because of the ability to transfer holes formed to electron-donating substrates by the cyanamide surface.^76^

### 3.1 Organic–inorganic hybrid materials (OIH)

**Fig. 6:**
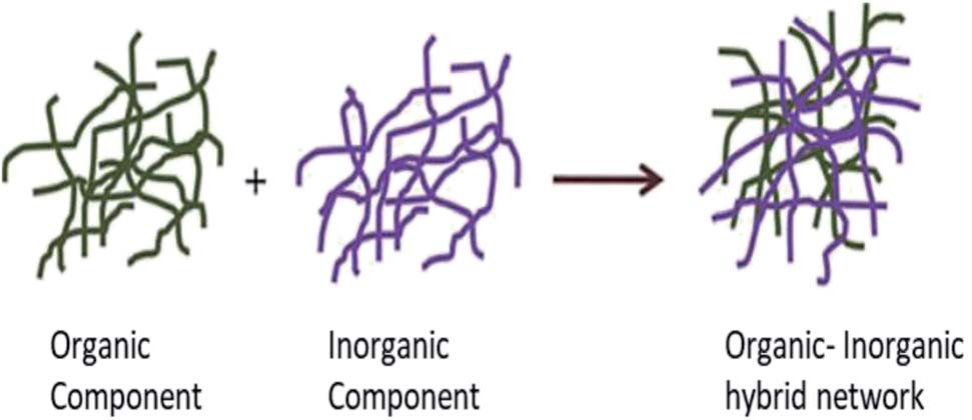
Representation of the interpenetrated organic–inorganic composites.^78^ Copyright 2019 Springer.

To improve the performance of conventional photocatalyst materials, organic–inorganic hybrid materials (OIH) have been deeply explored. The development and synthesis of novel catalysts having higher activity and versatile properties for photocatalytic applications are rapidly emerging in the research of material studies and industrial developments. Towards this objective, the flexibility to regulate the structural properties is an important criterion in the development of catalytic materials. If observed closely, nature has always followed a mechanism by which it combines materials even at lower scale, resulting in the formation of ‘‘hybrid materials’’. Bone and wood are examples of naturally occurring organic–inorganic composites. The former is composed of protein and minerals, and the latter contains cellulose fiber in a lignin matrix. Organic–inorganic hybrids, which showcase the features of both organic (variety, flexibility) and inorganic (conductivity, surface area) materials, evolves as a new division of photocatalytic material (Fig. 6). The concept of OIH material is basically the combination of organic and inorganic components into a single material. Integrating an inorganic material with organic molecules is an attractive pathway to manufacture functional hybrid materials with tunable properties through the systematic synthesis of hybrids of desired size and structure, and the tuning of optoelectronic properties that might improve the photocatalytic productivity.^77^ The advancement of inorganic–organic hybrid materials has inspired a large number of researchers towards this field. At the peripheral level, these hybrids will be either homogeneous or heterogeneous composites. The homogeneous arrangement consists of monomers and a miscible constituent, but the heterogeneous composite has a component with a dimension range between a few angstroms (Å) to nanometers (nm). OIH can be classified into two types, depending upon the type of interactions between the components.

In type 1, the physical interactions such as hydrogen bonding, van der Waals or ionic interactions are only allowed between the organic and inorganic components, which provide stability to the structure. Both organic and inorganic components contain some functional groups (polyamides, polycarbonates), which is responsible for the interaction. On the other hand, in type 2, chemical bonding such as covalent or non-covalent bond is responsible for the interaction between the two different phases. The miscibility between the two phases can be obtained by adding compatibilizers. Compatibilizers are macromolecules that are used to enhance the interfacial adhesion between both organic and inorganic components. These hybrid materials are promising towards catalytic applications.

**Fig. 7:**
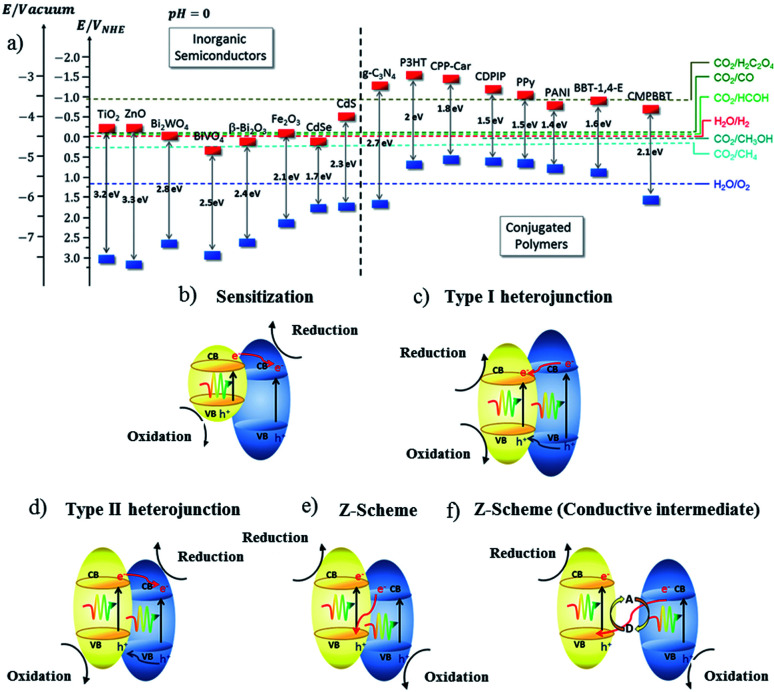
(a) Representation of the energy level diagram of a few inorganic semiconductors and conjugated polymers, and (b–f) the type of charge transfer mechanism in a hybrid semiconductor material.^79^ Copyright 2019, Chemical Society Reviews.

The development of photovoltaics is responsible for the evolution of photoconductive polymers and their widespread usage. The morphologies and properties of OIH materials can be adjusted on a large-scale parameter, and it can be reduced to very small sizes. The preparation of these materials is difficult due to its heterogeneous structures. So, it is highly essential and a tough task to design an OIH material with efficient photocatalytic activity. The initial works on these materials were based on inorganic semiconductors and organic components. The latter can either be molecular catalysts or dyes (organic), which can act as photocatalysts or sensitizers (Fig. 7). Organic macromolecular polymers can also be used due to its characteristics such as hydrophobic and hydrophilic nature. These polymers will not act as a photocatalyst, but can be protective films that aid the hybrid with their properties. Here, it is worth noting that the polymer is not considered as a part of the photocatalytic system. It just improves the activity of the entire system. When conjugated polymers are used, their conducting properties may contribute to the photocatalytic features. The delocalization of the alternating double and single bonds in the conjugated polymers are responsible for the photocatalytic properties. Recent literature has revealed that hybrid graphene–inorganic semiconductor materials have the potential to evolve as the modern material in the field of photocatalyst and composite industries.

### 3.2 Organic–inorganic hybrid materials for photocatalytic H_2_ production

#### 3.2.1 Carbon nitride materials for photo-reforming of biomass derivatives

**Fig. 8:**
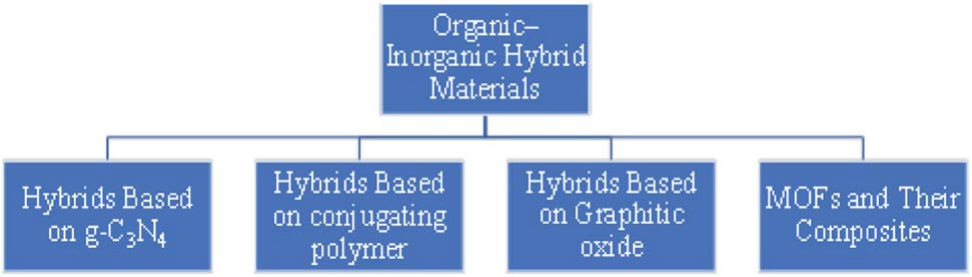
Organic–inorganic hybrid materials for photocatalytic H_2_ production.

**Fig. 9:**
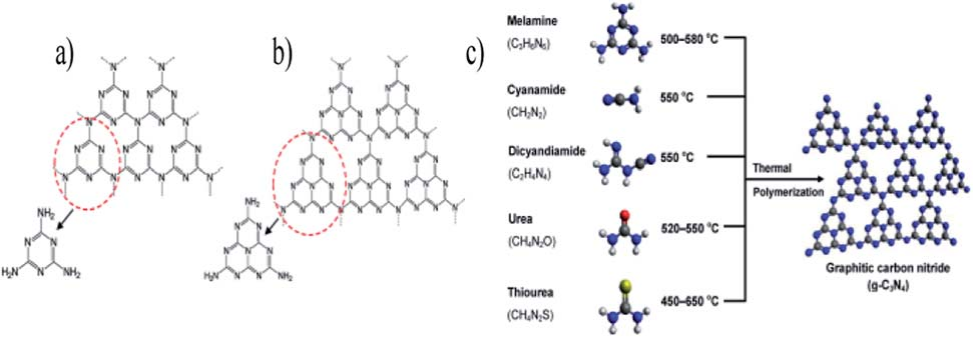
Structure of graphitic carbon nitride with (a) triazine, (b) tris-triazine, and (c) schematic representation of the preparation of g-C_3_N_4_ by the thermal polymerization of various precursors. The C, N, H, O, and S atoms are depicted by black, blue, white, red, and yellow balls, respectively.^83^ Copyright 2016, Chemical reviews.

The metal-free carbon nitride (C_3_N_4_) is a conjugated polymer that contains tertiary nitrogen's heptazine (C_6_N_7_) 2D-linked network units, and has evolved as a promising photocatalyst. Its characteristic properties include low preparation cost, high stability (550°, in both acid and base), synthesis from earth-abundant chemicals, unique band gap (*E*_CB_: −1.1 eV, *E*_VB_: +1.6 eV) and response to the 400–700 nm (visible) region. As g-C_3_N_4_ possesses these many magnificent features, it is a strong contestant for gaining the tag of ‘green photocatalyst’ compared to other semiconductors, such as CdS, BiVO_4_, and MoS_2_ by the scientific community. The availability of primary and secondary nitrogen at the edges and the hydrogen-bonded heptazine give many catalytically active centers for the biomass substrate. In recent studies, 2D carbon-containing materials have been widely used in biomass reforming, as they have good mobility of electrons, enhanced activity, large surface area and ability to form hybrid structures with other materials (Fig. 8). The adsorption of biomass onto the C_3_N_4_ sheets is facilitated due to the electron-rich 2D conjugated structure. The band gap of 2.7 eV makes it suitable for the visible light absorbing photocatalyst for biomass reforming. The synthesis strategies play a vital role in controlling the structure, which include regulating the size and morphology of the material. Two kinds of strategies have been carried for the synthesis of C_3_N_4_, which involves bottom-up and top-down approaches. The previous methods involved the use of a precursor containing a nitrogen compound with a template-assisted bulk g-C_3_N_4_ precursor under thermal oxidation or chemical exfoliation.^80^ Different strategies have been followed to control the morphology of the materials, such as the introduction of a co-catalyst, and the formation of a heterojunction with targets to improve light absorption.^81^ DFT data show that the valence band of g-C_3_N_4_ comprises the 2p state of nitrogen, and the conduction band consists of a hybridization of carbon 2p and the 2p state of nitrogen. So, the photogenerated holes will be present in the nitrogen sites. The photogenerated electrons suffer from the high recombination of the electron–hole pairs because of the hybridization of the nitrogen 2p and 2p carbon states in CB, which reduces the photocatalytic activity. Strong covalent bonding holds the carbon and nitrogen atoms together, forming layers of honeycomb-like structure. The van der Waals force between the layers of CN provides better stability in acidic or alkali solvents. C_3_N_4_ is synthesized from nitrogen-rich sources, such as melamine, urea, thiourea and dicyandiamide, through the process of thermal polycondensation and polyaddition (Fig. 9). More importantly, the polymeric nature of the material helps in achieving modulation and fictionalization at the molecular level. The synthesis of C_3_N_4_ hollow spheres with high crystallinity was achieved *via* molten salt method, where cyanuric acid–melamine was used as the precursor.^82^ The presence of heptazine units of CN served as a support matrix, which allowed it to merge with semiconductors, quantum dots and nanoparticles. Modification methods, such as nanostructure design, heterostructure construction, electronic structure modulation and loading of a cocatalyst, have been fruitfully illustrated in water splitting and biomass photo-reforming. A report showed that the lignocellulose photo-reforming exhibited a yield of 262 μmol h^−1^ g_cat_^−1^ with pristine polymeric carbon nitride.

**Fig. 10:**
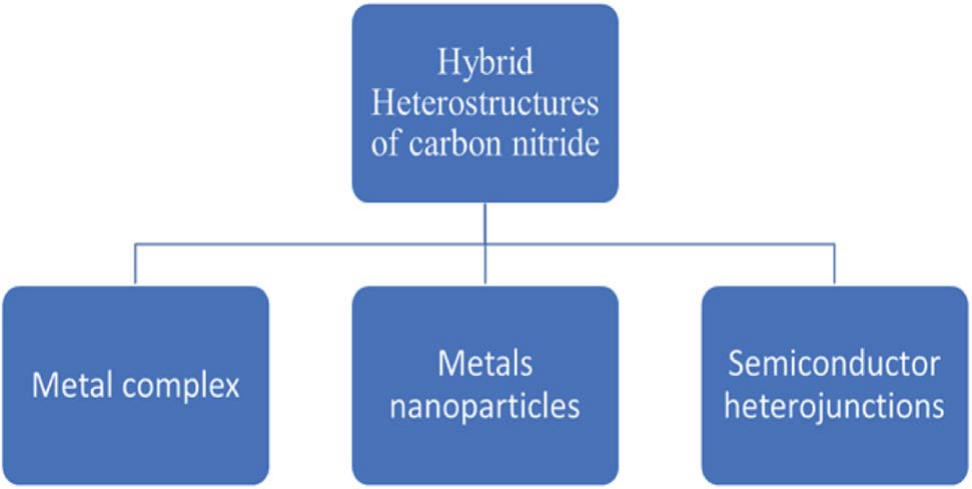
Different hybrid heterostructures of carbon nitride.

The mechanism is carried out by constructing carbon nitride curly sheets included on the surface in the plane heterostructure, which reduces the recombination of charge (charge separation) and optimization of the electronic band gap of the catalyst. The efficiency still improves to 4092 μmol h^−1^ g_cat_^−1^, where Pt (cocatalyst) is used. The same material is used for water splitting, which produces a yield of 22 043 μmol h^−1^ g_cat_^−1^ of hydrogen.^84^ The potential and efficiency of CN-based materials have been already well established in the field of photo-reforming of biomass. A further enhancement of activity is obtained by following certain modification techniques, such as surface functionalization, elemental doping, and construction of hybrid heterostructures (Fig. 10). Among these, the modifications through the modulation of the hybrid heterostructures will help to increase the photocatalytic activity of the material, as the recombination of the photogenerated electron–hole pairs are minimized. The incorporation of various composites will increase the biomass conversion efficiency. The use of metal complexes, metal nanoparticles, and semiconductors in hybrid carbon nitrides for successful hydrogen production from biomass will be briefly described.

The use of metals with g-C_3_N_4_ will enhance the hydrogen evolution reaction. The metal will use its surface plasmon resonance (SPR) phenomenon to increase the absorption of solar radiation with greater efficiency. The same metal can also play the role of a cocatalyst, which lowers the activation energy, thereby increasing the rate of hydrogen production in the presence of a scavenger (methanol, EDTA). Metals like Pt, Au, Ag, Rh and Ni are widely used as a cocatalyst to increase the efficiency of C_3_N_4_ for the hydrogen evolution. Jiang *et al.*(2019) synthesized a low-cost g-C_3_N_4_ surface modified using renewable bio-oil from pine saw dust. The oxygen-containing functional groups (C–O, C=O) helped in improving the separation of the holes and electrons, thereby enhancing the photocatalytic activity. The maximal yield of hydrogen obtained was about 1654 μmol h^−1^ g_cat_^−1^ at 180 °C.^85^ The currently used biomass sources like raw lignocellulosic materials have a few drawbacks, such as low solubility and inert behavior towards chemical reactions. These are avoided to an extent with the use of methods, like acid hydrolysis and ball milling.^86^ This review will be concentrated not only on raw biomass, but also on biomass-derived substrates, like alcohols, acids, aldehydes, and glucose, which are also feasible sources for hydrogen production. Industrial biomass waste products can be used as a source of hydrogen production substrate. The ethanol obtained from biomass sugar fermentation, glycerol produced from biofuels and methanol from the syngas are a few examples. Amines are biomass derivatives that have been receiving a lot of attention these days. Triethanolamine (TEOA) and triethylamine (TEA) are highly preferred in the H_2_ production, as they are used as scavengers in the reaction mechanism. Alcaudia *et al.*(2020) reported that g-C_3_N_4_ could be hybridized with TiO_2_ by hydrothermal method. The reaction was carried out using 3 wt% of triethanolamine, and the yield of hydrogen obtained was 1042 μmol g^−1^ h^−1^ with Pt as the cocatalyst. The formed heterojunction C_3_N_4_–TiO_2_ could give higher yield compared to pristine C_3_N_4_ and TiO_2_, respectively. This shows that the coupling of both materials could increase the spatial charge separation of the holes and electrons, and is supported by XPS data.^87^

**Fig. 11:**
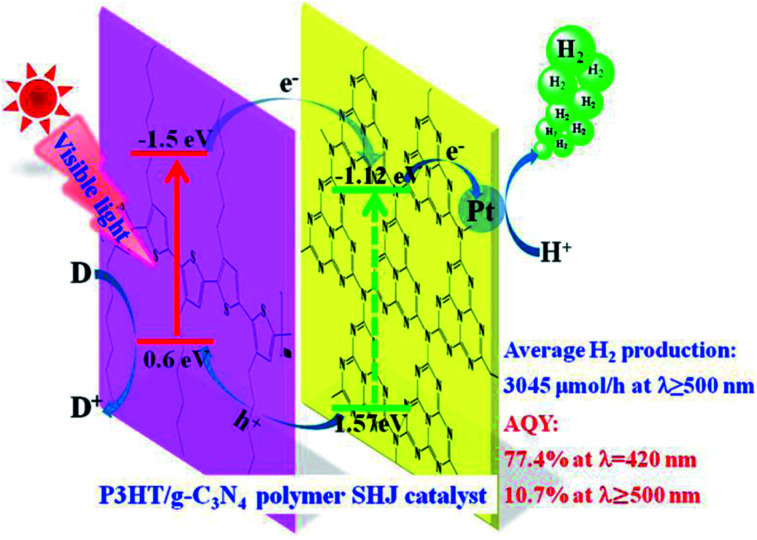
Plausible mechanism of hydrogen production using visible light over the surface heterojunction catalyst.^88^ Copyright 2019, International Journal of Hydrogen Energy.

Xiaohu *et al.* (2015) carried out hydrogen production using poly(3-hexylthiophene) in graphitic carbon nitride solution. The reaction yield varied when different sacrificial reagents were used. The surface heterojunction, which contained 3 wt% of P3HT, yielded 3045 μmol h^−1^ of hydrogen when ascorbic acid was utilized as a scavenger. The catalyst, when used with TEOA and EDTA, produces a yield of 320 μmol h^−1^ and 44 μmol h^−1^, respectively. The surface heterojunction catalysis mechanism is depicted in Fig. 11. The ability to absorb an extensive range of light and highly effective charge transfer among poly(3-hexylthiophene) and g-C_3_N_4_ is accountable for the efficient photocatalytic activity of the polymer–polymer catalyst. The efficiency of poly(3-hexylthiophene)–g-C_3_N_4_ decreases with higher composition of poly(3-hexylthiophene). This is attributed to two reasons: the hydrophobicity of P3HT is responsible for the agglomeration of the polymer, which makes it float above the solution surface and finally decreases the absorption of light. The other reason is that the activity of the co-catalyst is screened by the excess of P3HT, leading to decreased activity.^88^

Andera Speltini *et al.* (2018) described hydrogen production using aqueous biomass with stimulated light radiation. Oxidized graphitic carbon nitride (o-g-C_3_N_4_) is decorated with Pt, Cu–Ni co-catalysts in the presence of biomass and carbohydrates. The oxidized material (o-g-C_3_N_4_) with Cu–Ni cocatalysts yielded 912 μmol h^−1^. With Pt, the yield was 1170 μmol h^−1^. The usage of starch as the sacrificial agent has a large impact in the H_2_ evolution. The photoreaction basically involves the biomass acting as the scavenger of the oxidizing group, and metal acting as the proton reducing site. The potential uses of these low-cost materials can be used for the development of low environmental impact methods of hydrogen production.^89^

**Fig. 12:**
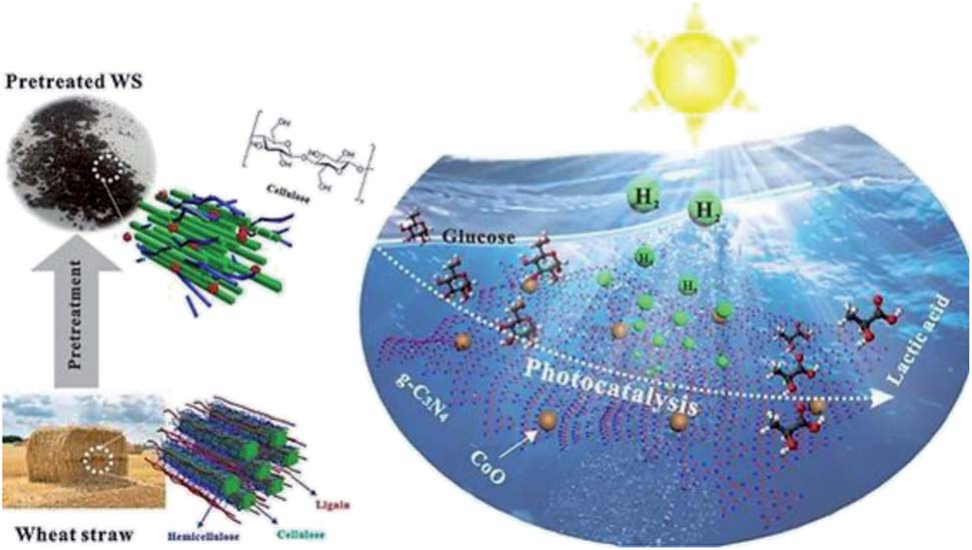
Schematic representation of the photo-reforming of wheat straw to produce hydrogen and lactic acid by photocatalysis.^50^ Copyright 2020, ACS.

Another report by Xinxing Wu *et al.* (2020) demonstrated an ideal path for the photocatalytic reforming with CoO/g-C_3_N_4_ for hydrogen evolution. The photo-reforming of phosphoric acid swollen cellulose (PASC) yielded 178 μmol h^−1^ g_cat_^−1^ of hydrogen. The higher activity was due to the interaction of PASC with the photocatalyst. This was confirmed by the quartz crystal microbalance analysis. The PR of raw lignocellulosic biomass wheat straw gives higher results on pre-treatment methods. The same study has shown an advantage of producing lactic acid, which provides value-added chemicals. So, this study widely opens the sunlight-driven biomass valorization to produce both hydrogen and value-added chemicals simultaneously (Fig. 12).^50^

**Fig. 13:**
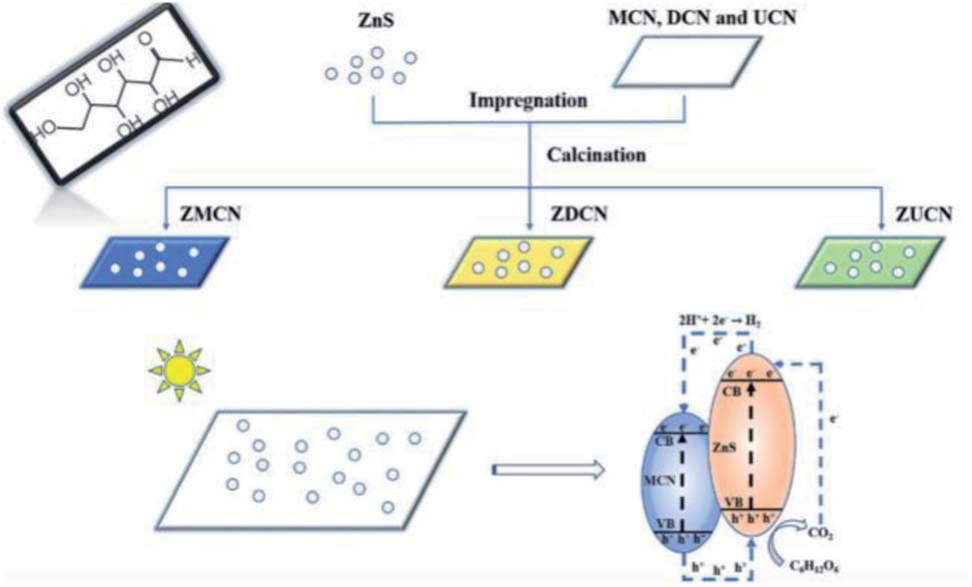
Mechanism of photocatalytic reforming of glucose on ZnS/C_3_N_4_.^90^ Copyright 2019, Journal of Colloid and Interface Science.

Xinyuan Xu *et al.* (2019) carried the photocatalytic hydrogen evolution using glucose as the substrate. In this study, ZnS nanoparticles (10–15 nm) were hybridized with g-C_3_N_4_ derived from various components, such as melamine, urea, and dicyandiamide. These two produce a heterojunction, which performs the reforming of biomass. The best yield was obtained from g-C_3_N_4_ produced from melamine. The hydrogen evolution reaction showed a productivity of 210 μmol g^−1^ with Pt as co-catalyst carried under 300 W xenon lamp light source. The electron and hole transfer mechanism of the photocatalyst is represented in Fig. 13. The photoluminescence spectra show that the introduction of ZnS with g-C_3_N_4_ reduced the charge carrier recombination. It is responsible for the enhanced hydrogen generation during the photoreforming of glucose.^90^

#### 3.2.2 Metal–organic framework-based photocatalysts for biomass photo-reforming

**Fig. 14:**
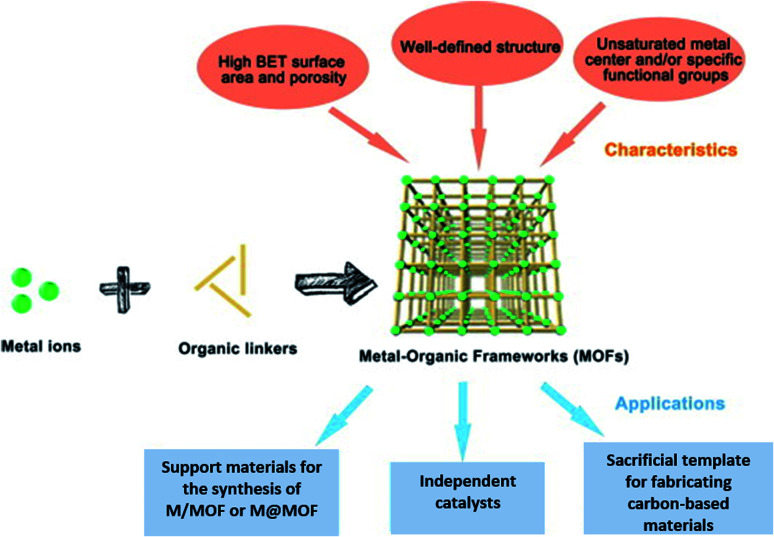
The components, structures, characteristics and applications of MOFs.^91^ Copyright 2018, Science Bulletin.

The valorization of the biomass to obtain fine chemicals and fuels has a diversified advantage compared to the fossil-based products. The utilization of metal–organic frameworks (MOFs) as a photocatalyst was explored in recent times for the photo-reforming mechanism. They are basically coordination polymers with a porous structure obtained by using organic ligands and metal atoms (Fig. 14). MOFs have advantages like a large surface area, tunability, and uniform porosity.

**Fig. 15:**
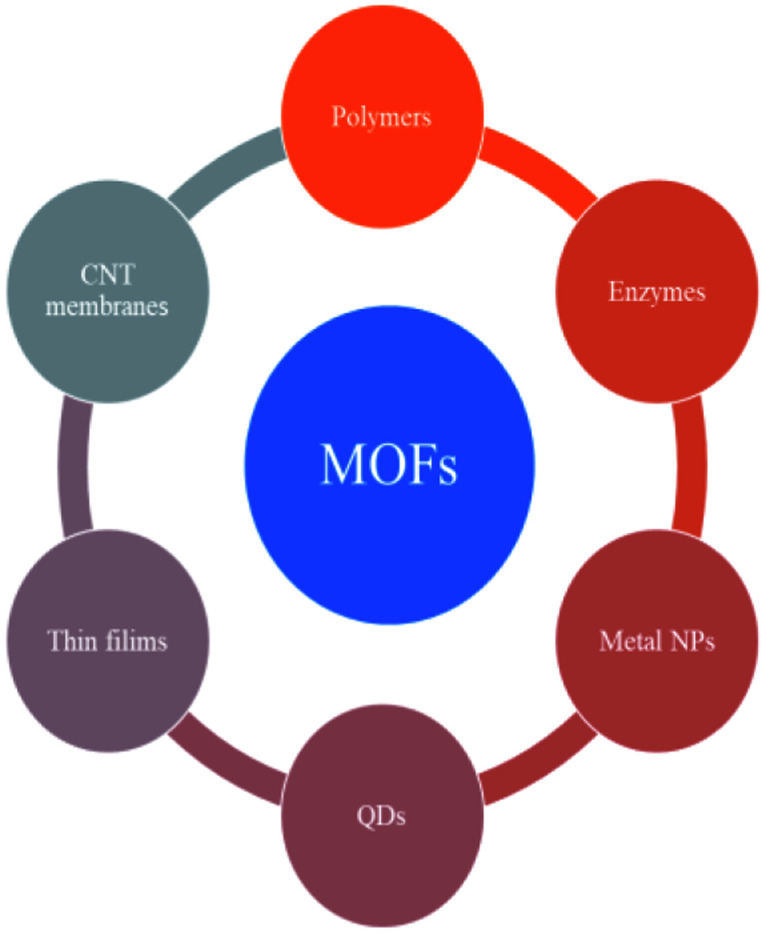
The composites of MOFs and functional materials.

**Fig. 16:**
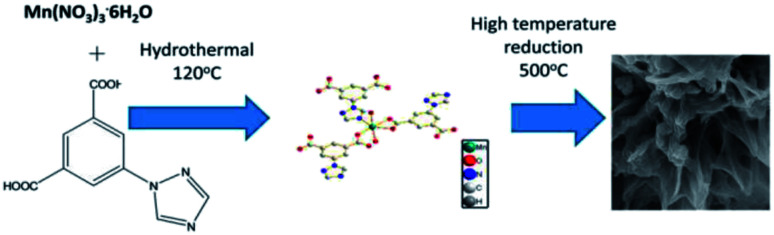
Mechanism of Mn–MOF–Au loading reduction at 500 °C.^94^ Copyright, 2018 Phase Transitions.

MOF composites can be prepared using metal nanoparticles, metal oxides, polymers, graphene, quantum dots, and carbon nanotubes (Fig. 15). Hydrogen production with MOFs is an ideal way for clean energy generation. Efficient hydrogen production from a water-containing sacrificial energy donor using visible light was achieved by metal-loaded MOFs. The water splitting mechanism is really a conventional way of hydrogen production, and is studied using MOFs to a larger extent. The Pt cocatalyst-incorporated MOFs, Zr_6_(μ_3_-O)_4_(μ_3_-OH)_4_(bpdc)_5.94_(L_1_)_0.06_ (ref. 92) and Zr_6_(μ_3_-O)_4_(μ_3_-OH)_4_(L_2_)_6_·64DMF,^93^ are efficient photocatalysts to obtain hydrogen fuel. This review emphasizes the methods by which hydrogen is produced by biomass and its derivatives. The hydrogen production from biomass substrates such as lignocellulose has not been explored much using metal–organic framework materials yet. Mingjin Luo *et al.* (2018)^94^ exploited photocatalysts based on metal–organic frameworks. In this study, the 2D MOF [M(TylP)_4_]_*n*_ (TylP = 5-(1,2,4-triazol-1-yl)isophthalic) was used as the photocatalyst material. Both Mn–MOF and Co–MOF were loaded with Au nanoparticles to carry out the photocatalytic reaction. The MOFs could be synthesized by a hydrothermal method at 120 °C followed by high temperature reduction (Fig. 16).

A turnover frequency of 576 μmol h^−1^ g_cat_^−1^ was calculated for the [Mn_2_(TylP)_4_] catalyst, which is higher than what is offered by the Co–MOF. An aqueous solution of triethylamine (TEA) was used as the scavenger. The study showed that upon adding the co-catalyst nanoparticle, the photocatalytic efficiency further increased. Thus, MOF as a heterogeneous catalyst opens a new arena for the hydrogen evolution reaction.^94^

**Fig. 17:**
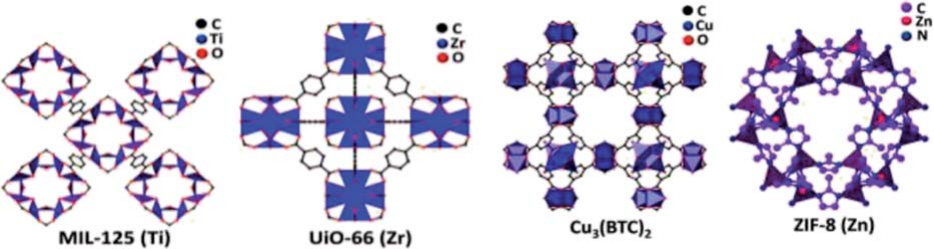
Structure of various MOFs (MIL-125(Ti), UiO-66 (Zr), Cu_3_(BTC)_2_, ZIF-8 (Zn)).^95^ Copyright 2019, ChemPhysChem.

The ease of tailoring the framework and the addition of functional components made MOFs competitive and promising photocatalysts in a large number of applications. The structure of a few MOFs is presented in Fig. 17. MOFs are extensively used in organic pollutant degradation, CO_2_ gas reduction, and metal reduction in wastewater. This implies that the photocatalytic activity of MOFs is more efficient than that of traditional semiconductor-based photocatalyst materials. The synthesis of MOFs is carried out through the liquid phase and solid phase reaction mechanisms. The latter is more simplistic and less time-consuming. The general methods that are largely used for the MOF synthesis are evaporation method, vapor diffusion method, gel crystallization, solvothermal, electrochemical and sonothermal methods. The mechanism and structure are the main factors that determine the photocatalytic efficiency of MOFs and other practical applications. The stability of the MOFs is often subjected to criticism as they are highly unstable in the aqueous conditions, which is considered as its major drawback. Even though MOFs are used to produce hydrogen from water, it has not been explored in the photoreforming of biomass to produce fuels. This application has a wide future scope, and is not yet fully explored.

**Fig. 18:**
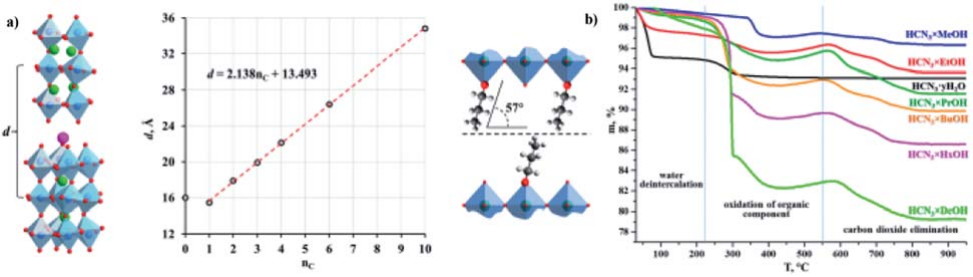
(a) Crystal structure, and their interlayer distance, (b) TGA curves of the niobates and *n*-alkoxy derivatives.^96^ Copyright 2021, Catalysts.

Vladimir V. *et al.* (2021) reported on hydrogen production using different organic–inorganic hybrid niobates Ca_2_Nb_3_O_10_-ROH, which contain *n*-alkoxy groups of primary alcohols inserted in the interlayer region. The photocatalytic hydrogen production is carried out using methanol solution using UV radiation. The activity was recorded for bare and Pt as the cocatalyst. The highest hydrogen evolution was produced by the ethoxy derivative Pt cocatalyst, HCa_2_Nb_3_O_10_EtOH/Pt, in different *n*-alkoxy derivatives. The apparent quantum efficiency in the 220–350 nm region is 20.6 percent. Using precalcined Nb_2_O_5_, CaO, and K_2_CO_3_ as starting materials, the alkaline layered perovskite-like niobate KCa_2_Nb_3_O_10_ (KCN_3_) could be created using the traditional ceramic process. The alkoxy derivatives (R) were synthesized by grafting the corresponding alcohols into a HCN_3_·*y*H_2_O interlayer space. The crystallinity of the samples and stability was confirmed by TGA (Fig. 18a and b). The organic alteration proved to be a successful method of increasing the niobate photocatalytic behavior for light-driven hydrogen evolution using aqueous methanol. The results indicate that the high efficiency of niobates for hydrogen generation from aqueous methanol using solar radiation has achieved because of the organic modification. The hybrid samples seem to be unstable under photocatalytic conditions, and the organic counterpart undergoes degradation during the reaction.^96^

**Fig. 19:**
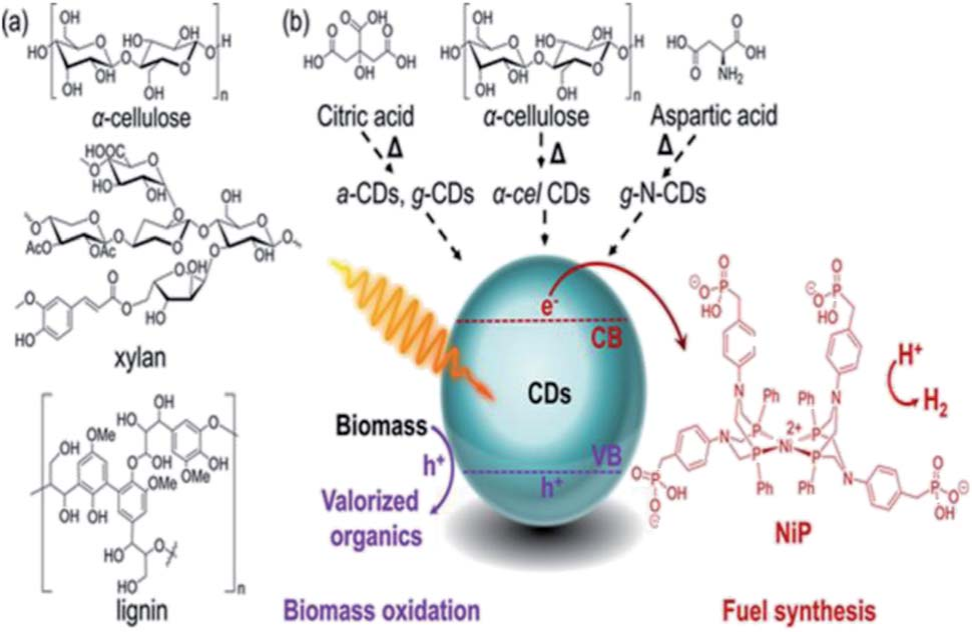
(a) Lignocellulosic component structures of electron donors. (b) CDs are prepared using biomass (α-cellulose) or commercial precursors (citric, aspartic acid), NiP as the cocatalyst in the PR of biomass to produce hydrogen.^97^ Copyright 2020, Angewandte Chemie.

Demetra S. Achilleos *et al.* (2020)^97^ reported a process that uses carbon dots (CDs) synthesized using the calcination of cellulose, and commercial precursors like aspartic acid or citric acid as the photocatalyst, which absorbs light for lignocellulose conversion into hydrogen and other organic components (Fig. 19). The photocatalytic system operates in ambient pH (2–8) at mild pressure and temperature. Carbon dots may be directly generated from biomass in a sustainable synthesis, and their solubility makes them compatible with insoluble biomass substrates.

**Fig. 20:**
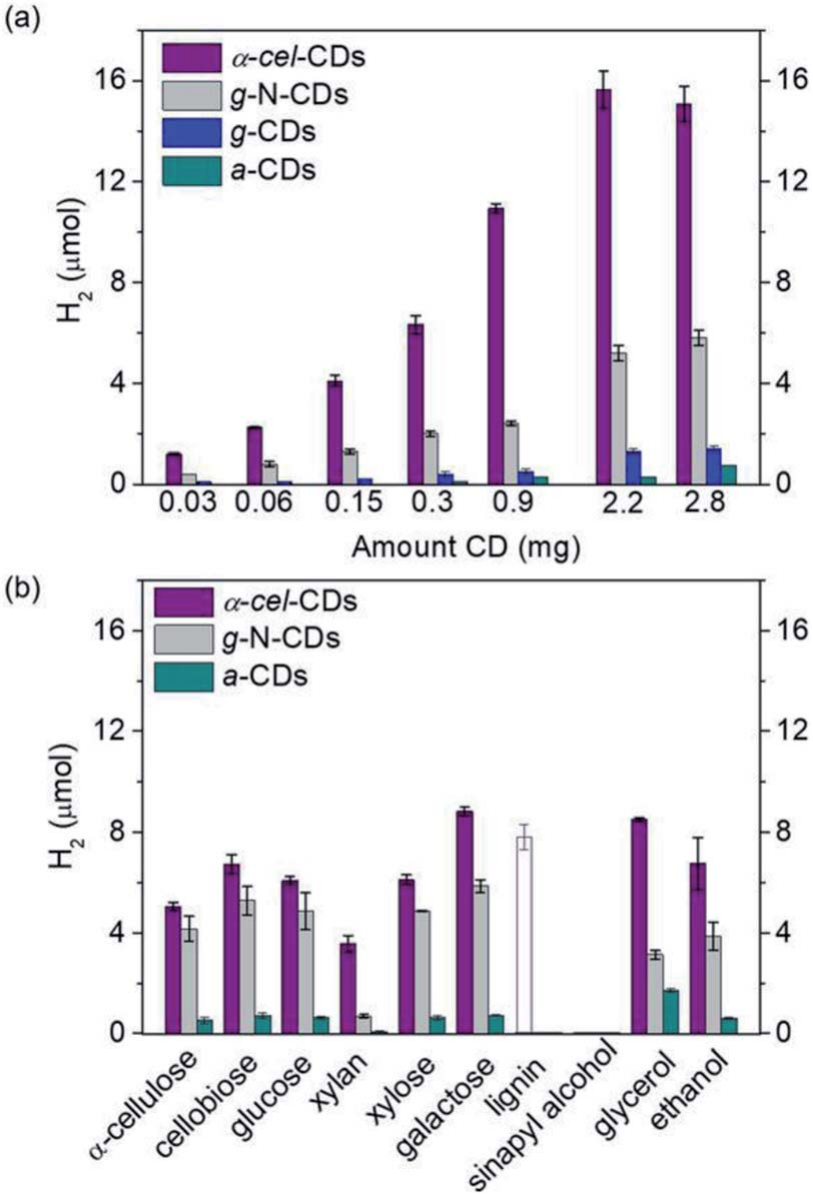
(a) Hydrogen production using α-cel carbon dots, g-N-carbon dots, g-carbon dots and α-carbon dots (0.03–2.8 mg), and EDTA (0.1 M, pH 6, 3 mL) as the sacrificial agent. (b) Hydrogen production with α-cell-carbon dots (2.2 mg), g-N-carbon dots (0.5 mg) and α-carbon dots (10 mg) with pure lignocellulosic components, and soluble substrates (100 mg, solid bars) in water (pH 6). The empty bar shows the result using 0.5 mg of lignin. The reaction was carried with NiP (50 nmol) for 24 h and 25 °C.^97^ Copyright 2020, Angewandte Chemie.

They also have good photophysical features, such as a large proportion of long-lived charge carriers and the ability to quench light both reductively and oxidatively. The homogeneous carbon dots are synthesized *via* scalable and controlled calcination of cellulose, or using citric acid and aspartic acid as commercial precursors for biomass PR. The nonpoisonous, compatible CDs and Ni bis(diphosphine) [cocatalyst] are used as light absorbers. The highest hydrogen production is shown by galactose (8.8 + 0.2 μmol) and glycerol (8.5 + 0.1 μmol) (Fig. 20)after 24 h reaction. The turnover numbers of NiP (TON) are 177 + 4 and 170 + 2, respectively. The EDTA is used as the sacrificial agent. These metal-free systems could preserve their photocatalytic activity even in untreated seawater. This opens up new possibilities for the creation of energy-independent and zero-carbon society.^97^

Beltram *et al.* (2016)^98^ showed the evolution of hydrogen from light and biomass derived alcohols using a hierarchical MWCNTs/Pd–TiO_2_ hybrid catalytic material. CNTs have been emerging as an active support in large number of catalytic reactions because of their optical, electronic, and thermal properties. The recent improvement includes the usage of titanium oxide and carbon nanotubes (CNTs) to produce an inorganic nanocarbon material with higher efficiency. In photocatalytic reactions, studies show CNTs as a good material that can take away the photoexcited electrons and reduce the recombination of charge, thereby increasing the speed of the reaction. The work was carried out using functional MWCNTs, Pd nanoparticles, and TiO_2_ on the photo-reforming of ethanol and glycerol using simulated sunlight radiation. The inorganic–nanocarbon hybrid was synthesized using benzoic acid functionalized MWCNTs, palladium nanoparticles with mercapto-undecanoic acid and titanium tetra (*n*-butoxide).

**Fig. 21:**
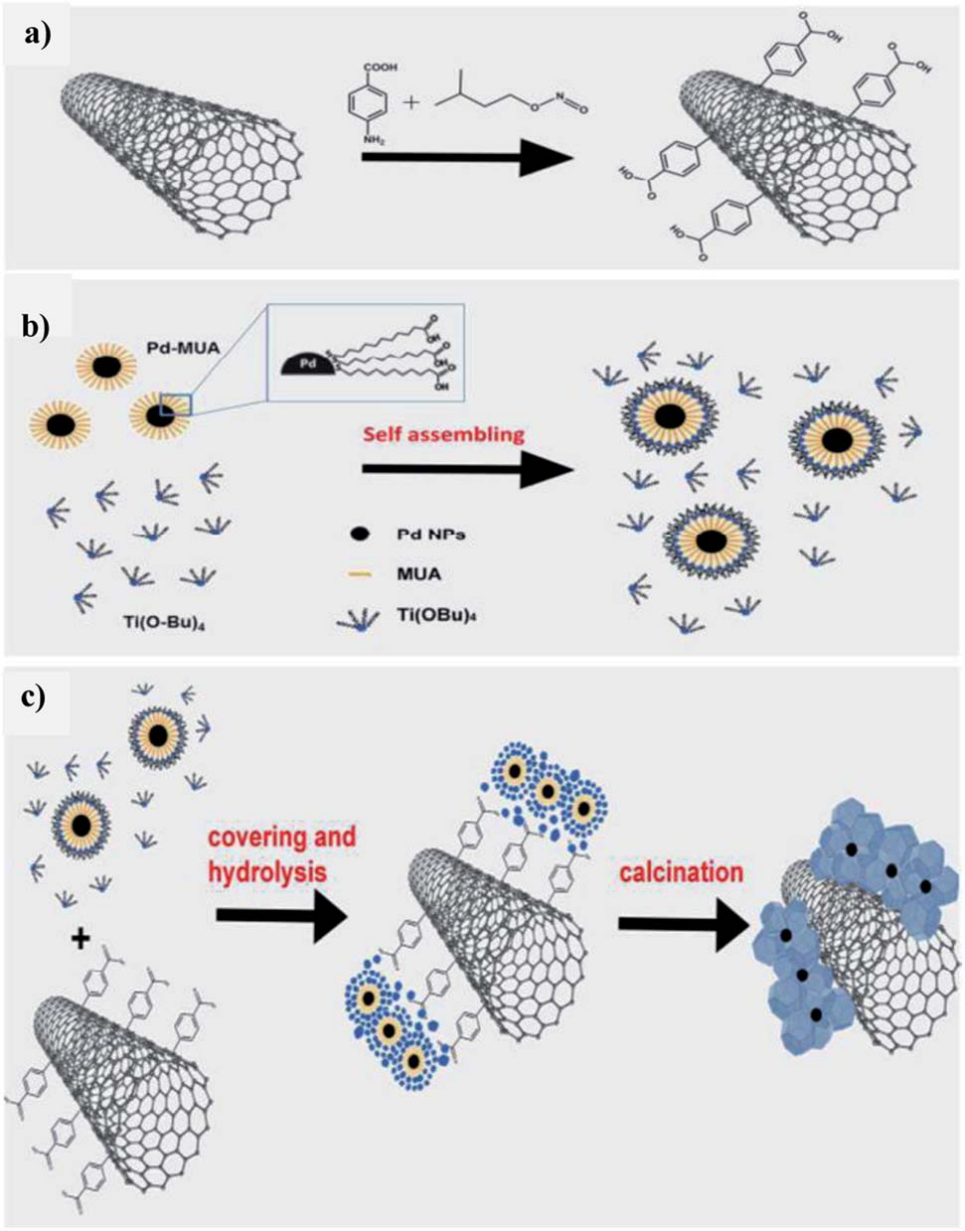
Schematic representation of hierarchical CNTs/Pd–TiO_2_ and CNTs/Pd–TiO_2_-calcination. (a) Covalent fictionalization of the MWCNTs with benzoic acid of *in situ* made diazonium salt *via* radical addition. (b) Pd–TiO_2_ precursor self-assembly mechanism. (c) Hydrolysis for obtaining the final material, fresh and calcined.^98^ Copyright 2016, Green Chemistry.

**Fig. 22:**
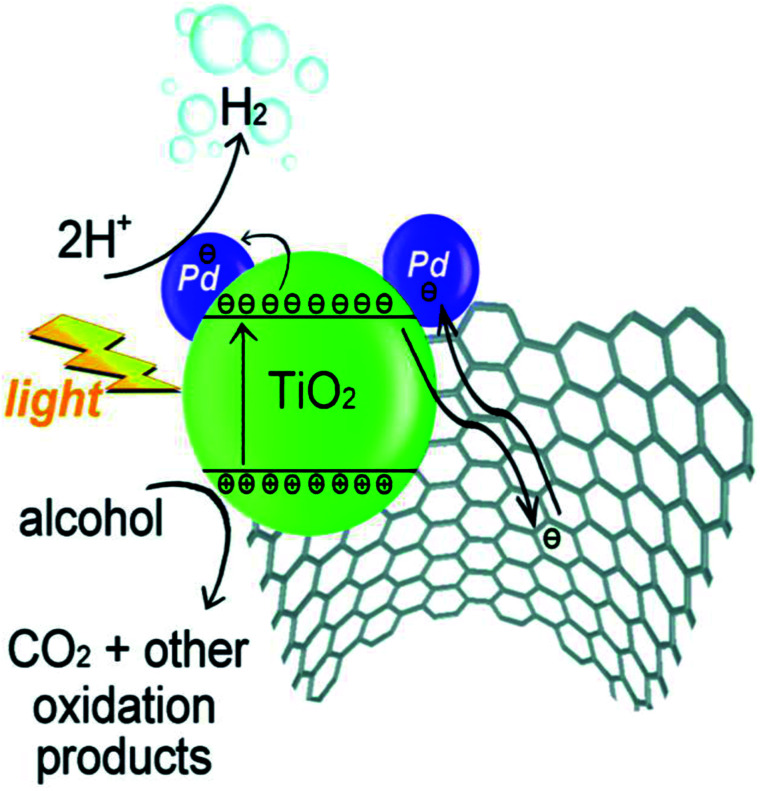
A proposed method for H_2_ synthesis with simultaneous scavenging of photogenerated holes by alcohol is schematically represented.^98^ Copyright 2016, Green Chemistry.

The *in situ* formed diazonium salt of benzoic acid was radically added, which was used to access nanotube blocks (Fig. 21a). A THF solution of Ti(*n*-OBu)_4_ was added to Pd–MUA to obtain Pd–TiO_2_ precursors (Fig. 21b). Hydrolysis with water or THF, followed by calcination, gives CNTs/Pd–TiO_2_ (Fig. 21c). The hydrogen yield reported when ethanol was used for 20-CNTs/Pd–TiO_2_ and 10-CNTs/Pd–TiO_2_ is 120 mmol g_cat_^−1^ h^−1^ and 14 mmol g_cat_^−1^ h^−1^ respectively. The quantum efficiency (QE) of 10-CNTs/Pd–TiO_2_ and 20-CNTs/Pd–TiO_2_ are 17% and 21%, respectively, at 365 nm. It shows decent photocatalytic efficiency of the material. In the case of glycerol, lower hydrogen production is observed in the case of both catalysts. The performance is 95 mmol g_cat_^−1^ h^−1^ and 149 mmol g_cat_^−1^ h^−1^, respectively. The hydrogen was produced in the reaction by H^+^ reduction by the electrons from the TiO_2_ conduction band, and are transferred to Pd nanoparticles in which they undergo further reaction (Fig. 22). The catalyst was found to be stable for over 24 h.^98^

**Fig. 23:**
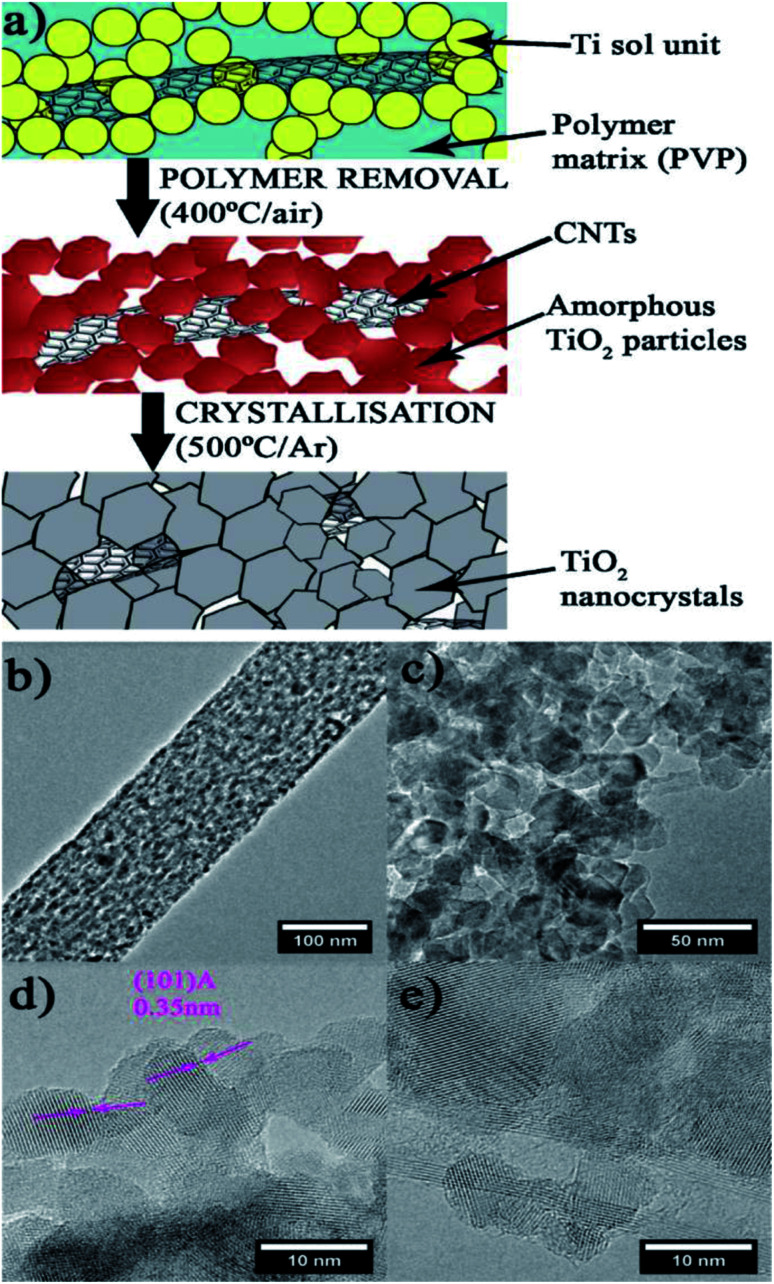
Scheme of the hybrid fiber for the synthesis. (a) Electrospinning: In a polymer fiber matrix, CNT and Ti precursors are incorporated (blue background). (b–e) TEM of the mesoporous structure produced by interlinked titania nanocrystals. (b) Polymer removal TiO_2_/CNT threads of linked TiO_2_ amorphous particles are formed by densification of the sol units. (c) Crystallization: generates titania nanocrystallites that have been hybridized with carbon nanotubes, resulting in mesoporous fibers. (d) Depiction of anatase's (1 0 1) lattice fringe and the tight interface across the nanocrystals of anatase. (e) CNT hybridized with nanocrystals of TiO_2_.^99^ Copyright 2015, Applied Catalysis B: Environmental.

Alicia Moya *et al.* (2015)^99^ reported on hydrogen evolution using a mesoporous CNT–TiO_2_ composite. The hybrid material synthesized by using electrospinning and sol–gel methods showcased a mesoporous structure of crystalline, interlinked inorganic nanoparticles (Fig. 23). This features a novel synthesis technique in which a large suitable polymer links the sol and the nanocarbons in the compound before calcination, and the polymer is removed by annealing the fibers in air, causing the sol to densify into a porous network of interconnected TiO_2_ particles. The photocatalytic activity is studied using ox-CNT–TiO_2_ material, electron spun TiO_2_ nanofibers and TiO_2_ nanoparticles, which produced hydrogen evolution values of 1218.5, 880 and 88.2 μmol h^−1^, respectively, using Pt as the cocatalyst under UV radiation. The oxygen vacancies and interface in the material have a crucial part in the structural evolution of the electron spun sample.

The sample shows higher activity due to the presence of oxygen vacancies. The catalyst has a mesoporous structure of interlinked nanocrystals with a large TiO_2_–TiO_2_ interface to enhance charge transfer. Furthermore, the CNTs were hybridized with TiO_2_ nanocrystals to form a metal semiconductor junction with CNTs acting as electron acceptors, which improves charge separation and stability ^99^

### 3.3 Biomass derived substrates

The hydrogen production from water splitting in ambient conditions using solar radiation is considered as the cheapest, efficient and eco-friendly means of fuel production. On the other hand, the biomass forms an alternative substrate for the photoreduction to form hydrogen with the addition of value-added chemicals. The use of raw material biomass is hardly explored in photocatalytic hydrogen production, but biomass derivatives are widely used as substrates for the same purpose. The remaining part of the review will focus on the feasibility and potential of biomass derivatives such as monomeric substrates for the photocatalytic hydrogen synthesis using OIH materials. Numerous monomeric substrates from industrial and agricultural processing can be used to serve as the feedstock of the photoreforming mechanism. This mainly includes glycerol, methanol and ethanol, which is produced *via* different mechanisms such as biofuel production, syngas production and sugar fermentation, respectively. The future objective of the scientist community is to head towards a green chemistry approach, where the reaction substrates are expected to be produced from non-energy intensive sources or non-fossil fuels. The substrates that are most reliable and promising for the photocatalytic hydrogen production are aldehydes, alcohols, amines and acids. The use of sacrificial agents is widely used in the photocatalytic hydrogen productions in aqueous solutions. The works on photocatalytic hydrogen production is commonly mentioned *via* a generic term ‘water splitting’ in publications, which will be carried out by methanol/ethanol or other biomass derivative as a sacrificial agents in an aqueous media for the reaction. Even though greater efficiency has been obtained in the field of photoreforming, this area has not really been explored as a source of hydrogen production. The use of biomass derivatives as substrates in the photoreforming mechanism will be briefly analyzed in the following section.

**Fig. 24:**
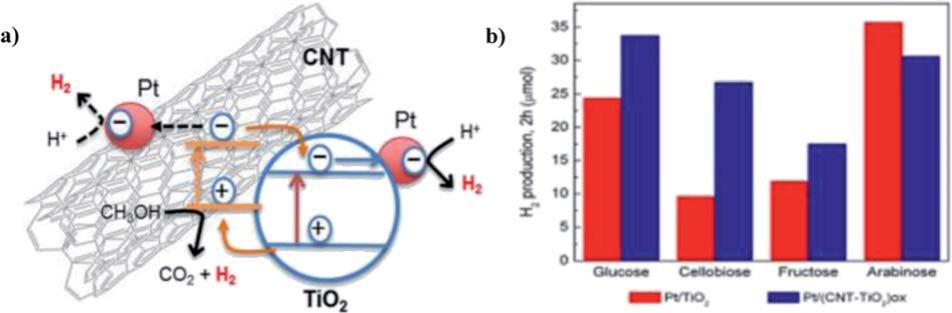
(a) Scheme for the photocatalytic mechanism of H_2_ generation from water/methanol solutions by the Pt/(CNT–TiO_2_)ox-473 catalyst using near UV-visible radiation. (b) H_2_ produced from saccharides (0.02 M) with Pt/TiO_2_ and Pt/(CNT–TiO_2_)ox catalysts.^100^ Copyright 2015, Applied Catalysis B: Environmental.

Claudia G. Silva *et al.* (2014) reported on the photocatalytic hydrogen production from methanol and saccharides using carbon nanotubes–TiO_2_ catalysts. The CNT–TiO_2_ catalyst was synthesized using a composite procedure. The one-pot method consisting of the oxidation of CNT and preparation of the composite leads to the development of materials having efficient productivity due to the good interaction between TiO_2_ and the CNT interface. The incipient wetness method is used to incorporate Pt, Au, Pd and Ir nanoparticles in (TiO_2_ and CNT–TiO_2_)ox. The activity is higher in the metal-loaded catalysts than those non-loaded catalysts. This can be explained by the low overpotential for hydrogen production on the metal-added photocatalyst than the bare catalysts. The material treated at higher temperature (673 K) showed higher efficiency than those at low temperature (473 K) because the metal particles were sintered and the metal support interface was stronger. The highest yield was obtained for the Pt/(CNT–TiO_2_)ox catalyst for hydrogen production of 30.6 μmol in water/methanol solutions (Fig. 24). Saccharides are effective sacrificial donors for HER. It was observed that the efficiency of hydrogen generation with Pt/(CNT–TiO_2_)ox increased with the decreasing complex molecular structure of saccharides. The electrons from TiO_2_ and CNT are expected to be photoexcited. Electrons from VB are excited to the CB of TiO_2_, and then transferred to Pt nanoparticles to generate H_2_. The high contribution of water proton reduction on the metal particle surface is credited with superior activity in the case of fructose, glucose and cellulobiose solutions.^100^

**Fig. 25:**
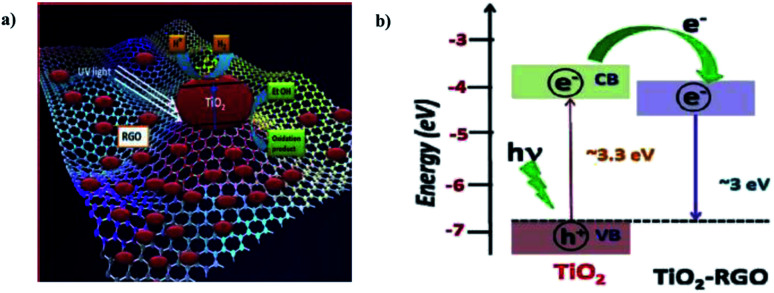
(a) Representation of the photocatalytic hydrogen production from TiO_2_–RGO-based photocatalyst mechanism. (b) Energy level diagram of TiO_2_ and RGO.^101^ Copyright 2015, International Journal of Hydrogen Energy.

G. Nagaraju *et al.* (2015) reported on photocatalytic water splitting using a TiO_2_-reduced graphene oxide (RGO) photocatalyst *via* ionothermal method by functionalist ionic liquid. The characterization data of the composite proved that the TiO_2_ nanoparticles were placed onto the graphene sheet surface (Fig. 25). The reaction was carried out in water and ethanol solution using TiO_2_–RGO composites. The hydrogen yield was given as 3.0 mmol g^−1^ through water splitting, which is more than the performance of the pristine TiO_2_ particles. The yield enhancement is described using the synergic effect of reduced graphene oxide.

The significant photocatalytic activity of graphene is due to trap an excesses electrons and minimizing the electron–hole pair recombination under light illumination.^101^

**Fig. 26:**
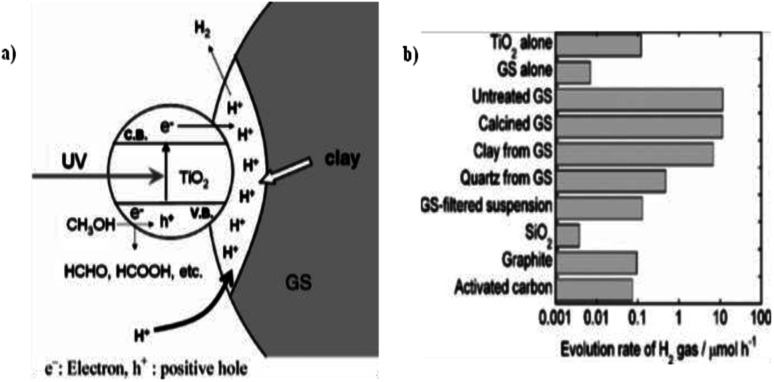
(a) Photocatalytic reduction of hydrogen ions contained in the clay in GS increases hydrogen generation. (b) Hydrogen evolution comparison. Powdered TiO_2_ (15 mg) is added with each powdered additive (15 mg). For TiO_2_ alone and GS-alone systems, 30 mg was taken, 40% vol. of methanol.^102^ Copyright 2006, Journal of Photochemistry and Photobiology A: Chemistry.

Miyuki Ikeda *et al.* (2006) reported on the hydrogen evolution using water and methanol mixture with mixture of TiO_2_ and graphitic silica. A synergic effect on the production of hydrogen was obtained from GS and TiO_2_ from water–methanol mixtures. This synergic effect was studied by evaluating the hydrogen evolution with different composites of TiO_2_ using bare GS, SiO_2_, calcined GS, clay from GS, activated carbon and quartz from GS. It was found that the synergic effect is due to an increase of hydrogen ions in the clay components of GS, and accumulation of GS and TiO_2_ (Fig. 26a). The highest rate of hydrogen production is reported as 22.3 μmol h^−1^, and this rate has been obtained at a GS composition of 50 wt%. However, pure TiO_2_ or GS give just 0.23 and 0.03 μmol h^−1^ (Fig. 26b), respectively.^102^

Sundaram Ganesh Babu *et al.* (2015)^103^ reported on the scalable and economic synthesis of reduced graphene oxide (RGO) with the Cu_2_O–TiO_2_ composite (surfactant free) catalyst *via* wet impregnation method aided by ultrasound. The UV-DRS spectroscopical studies show that the incorporation of Cu_2_O brings a change in the band gap from 3.21 eV to 2.87 eV. The HER was studied in the presence of glycerol (sacrificial agent) under visible light.

**Fig. 27:**
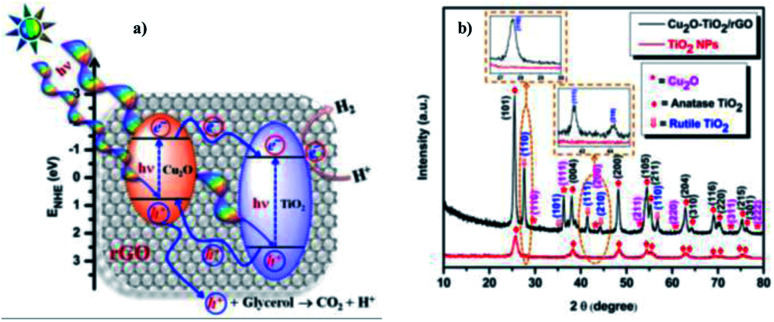
(a) Representation of the Cu_2_O–TiO_2_/RGO water splitting reaction mechanism. (b) X-ray diffraction data of pristine TiO_2_ nanoparticles and Cu_2_O–TiO_2_/RGO photocatalysts.^103^ Copyright 2015, Nanoscale.

The incorporation of RGO increases the mobility at the Cu_2_O–TiO_2_/RGO photocatalyst p–n junction, and this is evident from the decreased luminescence intensity of the Cu_2_O–TiO_2_/RGO material. Based on the energy and position of the VB and CB of Cu_2_O, a mechanism of hydrogen production is shown in Fig. 27a. The crystallinity and purity of the sample were examined through XRD analysis (Fig. 27b). The hydrogen evolution rate was 110 968 μmol g^−1^ h^−1^ when 1.0% Cu and 3.0% graphitic oxide were used, which is more than that of the formerly studied graphene-based photocatalyst. The RGO enhanced the efficiency of the photocatalyst compared to bare TiO_2_ and Cu_2_O–TiO_2_ by a factor of 14% and 7%, respectively.

**Fig. 28:**
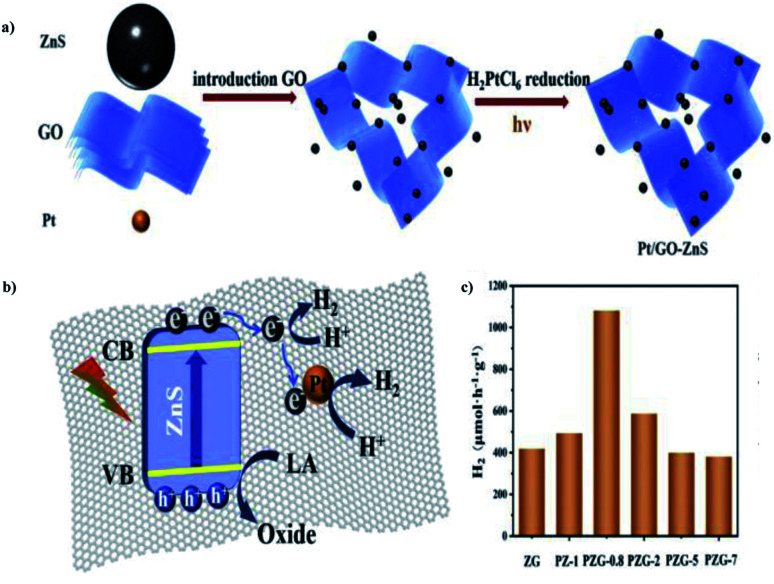
(a) Scheme for the preparation of Pt/GO–ZnS through the stepwise mechanism. (b) Schematic representation of the structural benefits of the Pt/GO–ZnS for HER. (c) Hydrogen production using different photocatalysts.^104^ Copyright 2020, International Journal of Hydrogen Energy.

Luna Tie *et al.* (2020) constructed a photocatalyst with graphene oxide in the microsphere ZnS, which delivered more productive hydrogen evolution. The preparation of the Pt/GO–ZnS photocatalyst could be achieved *via* two steps: the hydrothermal synthesis of ZnS, followed by the deposition of GO and Pt through *in situ* mechanism. The combination of ZnS with GO and Pt nanoparticles is a stepwise deposition process, as described in Fig. 28a. HER was carried out *via* water splitting with lactic acid as the scavenger using 300 W Xe lamp. The results show that the incorporation of GO sheets and Pt nanoparticles improved the efficiency of the catalyst. The improved yield of Pt/GO–ZnS was because of the efficient charge separation and higher visible range absorption. This is credited to the synergetic effects of GO and Pt nanoparticles. The excited electrons in the CB of ZnS were trapped instantly by GO, and transferred to the Pt nanoparticles. The tight interface is responsible for the smooth mechanism (Fig. 28b). The catalyst exhibited a yield of 1082 μmol g^−1^ h^−1^ of hydrogen, which is much higher than that of the pristine ZnS catalyst (Fig. 28c).^104^

**Fig. 29:**
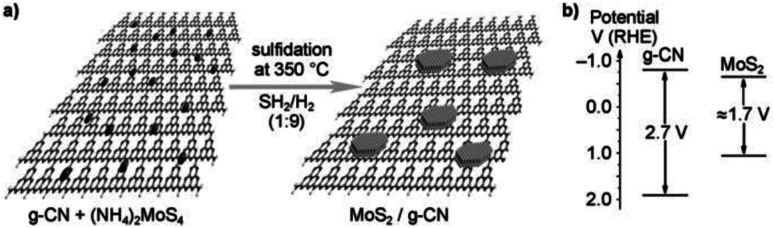
(a) The theoretical structural model of the resultant MoS_2_/g-carbon nitride layered junctions and the process for gas-phase sulfidation. (b) g-CN and MoS_2_ band gap energy diagram.^105^ Copyright 2013, Angewandte Chemie.

Yidong Hou *et al.* (2013)^105^ carried out the hydrogen evolution reaction using MoS_2_ with the mesoporous graphitic carbon nitride nanojunction. The construction of OIH with MoS_2_ and the g-CN heterojunction was carried out *via in situ* growth method, which involves both impregnation and sulfidation (Fig. 29a). The structure of MoS_2_ is similar to that of graphite. This will minimize the lattice difference, and MoS_2_ can be easily grown above the g-CN surface. This kind of arrangement has a few benefits, such as increasing the accessible space about the interface and reducing the electron transport barrier through the cocatalyst.

This aids in the fast electron transport through the interface *via* the electron tunnelling effect. The reaction proceeded with the MoS_2_/mpg-CN photocatalyst and lactic acid as the scavenger under visible radiation. The MoS_2_/mpg-CN nanojunction shows an HER of 20.6 μmol h^−1^, which is more than the yield offered by Pt/mpg-CN at 4.8 μmol h^−1^. This study evaluates the design of an effective and thin interface 2D nanojunction between the cocatalyst and semiconductor having similar geometry.^105^

**Fig. 30:**
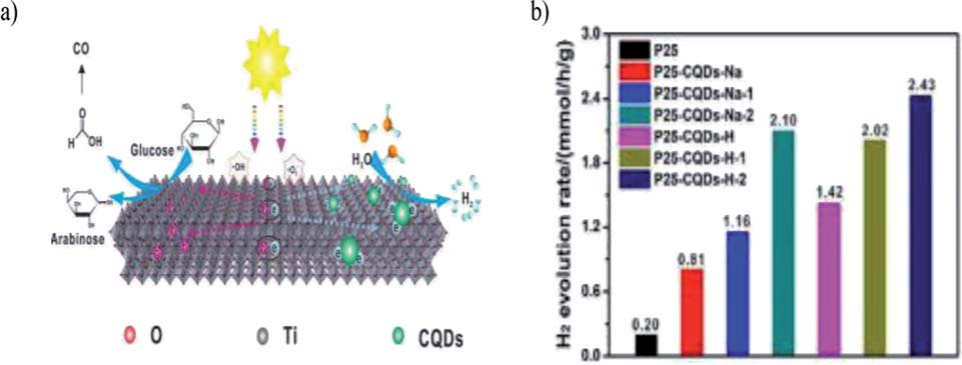
(a) Illustration of the photoreforming reaction mechanism. (b) Photocatalyzed hydrogen evolution production for the various composites.

Heng Zhao *et al.* (2022) reported on the production of hydrogen using a composite of carbon quantum dots modified with TiO_2_. This work focused on the photoreform of glucose for producing hydrogen under ambient conditions. The synthesized CQD–TiO_2_ facile one step hydrothermal method used sodium citrate and citric acid as the source for carbon. The CQDs were evenly distributed on the TiO_2_ surface. The photocatalytic activity was carried out with composites having carbon dots of different sources, and the highest hydrogen production yield of 2.43 mmol h^−1^ was obtained with methanol as the sacrificial agent. The heterojunction formed between the rutile and anatase phase aided in the separation of the photogenerated charge carriers. Fig. 30a depicts the proposed reaction mechanism of photoreforming. This works depicts an innovative strategy to control the carbon quantum dot particle size to carry out metal-free photocatalysis.^106^ Hydrogen production using biomass with organic–inorganic hybrid materials and its derivatives are represented in Table 1.

**Table 1:** Organic–inorganic hybrid photocatalytst for hydrogen production using biomass and its derivatives

S. no.	Photocatalyst	Substrate	Light source	Reaction solution	H_2_ production rate	AQE	Ref.
1	Pt/C_3_N_4_–TiO_2_	Triethanolamine	250 W, visible light radiation	10 wt% of TEOA + 3 wt% Pt	1042 μmol g^−1^ h^−1^	—	87
2	Poly(3-hexylthiophene)/g-C_3_N_4_	Triethanolamine	300 W, Xe lamp 420 nm	3 wt% catalyst + 1 wt% Pt	320 μmol h^−1^	—	88
3	Poly(3-hexylthiophene)/g-C_3_N_4_	Ethylenediamine tetra-acetic acid	300 W, Xe lamp 420 nm	3 wt% catalyst + 1 wt% Pt	44 μmol h^−1^	—	88
4	Poly(3-hexylthiophene)/g-C_3_N_4_	Ascorbic acid	300 W, Xe lamp 500 nm	3 wt% catalyst + 1 wt% Pt	3045 μmol h^−1^	59.4%	88
5	Pt/Holey carbon nitride–*N*-acetylethanolamine (HCN–NEA)	Triethanolamine	300 W, Xe lamp 420 nm	0.8 wt% Pt, 30 mg catalyst	22 043 μmol g^−1^ h^−1^	41.2%	84
6	Cu–Ni/o-g-C_3_N_4_	Brewery/dairy effluent 50	Solar simulator	1–5 wt% Cu–Ni + starch solution	912 μmol g^−1^ h^−1^	—	89
7	Pt/o–g-C_3_N_4_	Brewery/dairy effluent 50	Solar simulator	3 wt% Pt + starch solution	1170 μmol g^−1^ h^−1^	—	89
8	CoO/g-C_3_N_4_	Wheat straw	300 W, Xe lamp	1.7 wt% Co + cellulose	178 μmol g^−1^ h^−1^	—	50
9	ZnS–C_3_N_4_	Glucose	300 W, Xe lamp	1 wt% Pt + glucose	210 μmol g^−1^ h^−1^	—	90
10	Mn–MOF–Au	TEA	300 W, Xe lamp	Pt + TEA	576 μmol g^−1^ h^−1^	—	94
11	HCa_2_Nb_3_O_10_–ROH	Methanol	125 W, Hg tube lamp	Pt + 1 wt% methanol	500 μmol g^−1^ h^−1^	20.6%	96
12	Cellulose–CDs	Lignocellulose	—	NiP + 0.1 M EDTA	13 450 μmol g^−1^ h^−1^	11.4%	97
13	MWCNTs/Pd–TiO_2_	Ethanol	150 W, Xe lamp	Pd + ethanol	120 mmol g^−1^ h^−1^	21%	98
14	oxCNT–TiO_2_	Methanol	200 W, Hg tube lamp	0.5 wt% Pt + methanol	1218 mmol g^−1^ h^−1^	—	99
15	oxCNT–TiO_2_	Saccharides	—	Pt + methanol	30.6 μmol	—	100
16	TiO_2_–RGO	Ethanol	—	Water + ethanol	3.0 mmol g^−1^	—	101
17	TiO_2_–graphitic silica	Methanol	—	Methanol + water	22.3 μmol h^−1^	—	102
18	Cu_2_O–TiO_2_/RGO	Glycerol	250 W, Xe lamp	Glycerol + water	110 968 μmol g^−1^ h^−1^	—	103
19	GO–ZnS	Lactic acid	300 W, Xe lamp	Lactic acid + water	1082 μmol g^−1^ h^−1^	—	104
20	MoS_2_/mpg-CN	Lactic acid	—	Lactic acid + water	20.6 μmol h^−1^	—	105
21	CDs–TiO_2_	Methanol	300 W, Xe lamp	Methanol + water	2.43 mmol h^−1^		106

## 4. Perspective and conclusions

The article deals with the recent developments and scopes of organic–inorganic hybrid (OIH) materials in the photo-reforming of biomass and its derivatives. The unavailability of efficient and stable photocatalytic materials for various applications in this research area is a major barrier for the innovative and effective research work. Although different types of photocatalytic materials, such as inorganic semiconductors, metal oxides, sulphides, and polymers, are widely used in the hydrogen evolution reaction, the class of organic–inorganic hybrid materials has been deeply studied in the last few decades. The material used for photocatalytic reactions should possess certain features, such as abundant active sites, high surface area, good charge transfer and band structure tunability for the efficient productivity of hydrogen. The class of OIH material is gaining good attention in the photocatalytic studies due to its tailor-made synthesis mechanisms, band gap tunability, absorption range, stretchability, and charge transfer ability. These materials can enhance the physiochemical properties and enhance the hydrogen production rate by increasing the active areas, bringing a change to the activation energy of the reaction, and finally bringing an optimization in the catalytic performance. These materials can be used as is or combined with others as a protective counterpart or dye sensitizer, leading to the formation of hybrid heterojunctions. The review gives the attention to the ongoing trends and advanced developments in the OIH materials for hydrogen evolution from biomass & its derivatives.

**Fig. 31:**
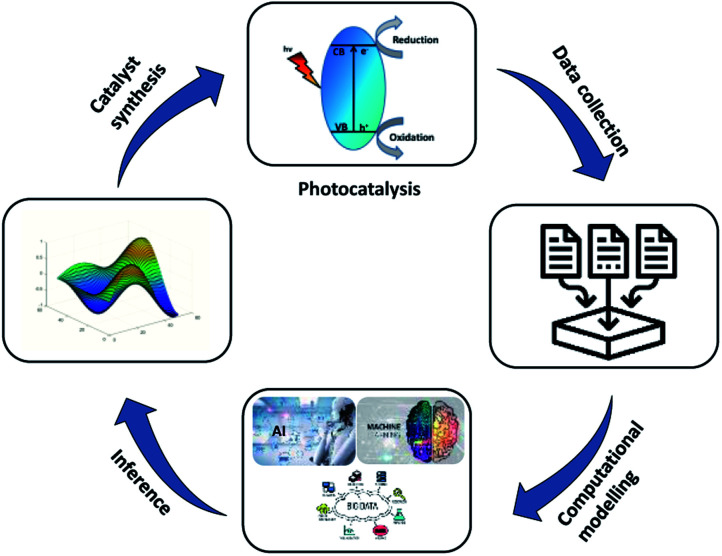
Graphical representation of the photocatalyst with computational modelling.

It is necessary to employ appropriate theoretical techniques with experimental analysis in order to construct photo catalytically active and efficient devices. The computational analysis techniques have been an emerging technology in the field of photocatalysis research, which can further provide a deep understanding of the photocatalytic materials. Computational studies can give valuable details regarding reactivities and properties of the material based on their electronic structure using quantum mechanical and molecular mechanical studies. Density functional theory (DFT), machine Learning, artificial intelligence and big data analysis are emerging technologies that can provide mechanistic properties about the material, and aid us in the design of more stable and efficient photocatalysts (Fig. 31).

**Fig. 32:**
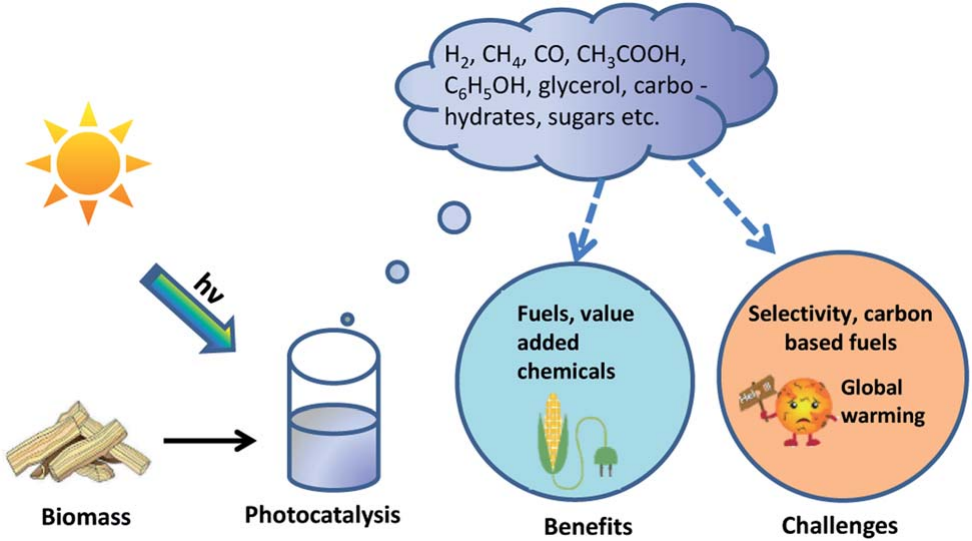
Schematic illustration of the biomass photo-reforming, benefits and challenges.

DFT studies basically a quantum mechanical model which can be used to study band structure, charge density and band structure of the photocatalyst.^107,108^ The accurate results regarding the material properties can be obtained by increasing the thickness of the material slab in the simulations, resulting in increasing the computational cost.^109^ Machine learning (ML) is also one of the developing technologies, which used computational algorithms to convert empirical data to useable models.^110^ The coupling of the photocatalysis domain with ML can pave a way for the growth of catalyst materials. The development of ML models with the existing ideas can lead to a pathway to find efficient photocatalysts. It involves the integration of prior theoretical and empirical knowledge to models, and uses the available data to predict the results. The shortage of reliable data and difficulty in collecting new data remain the main challenges in ML.^111,112^ Big data analysis is also a similar method, which uses bibliometric reports on a particular research topic and provides scientific and theoretical information to the scientific community.^113^ Information regarding the results of previous studies conducted in a particular topic is collected and combined as a database.^114^ The difficulty in finding a credible and authentic data has the main issue related to big data analysis. Recently, artificial intelligence (AI) also came into this scenario for the prediction and stimulation of photocatalyst materials. This can be used to identify various interactive influences of parameters, and finding the optimum strategy to obtain the maximum efficiency of the material. The ability of AI to predict without having perfect and complete information and pattern recognition ability can be widely explored the field of research.^115^ All of the above mentioned computational methods are in the initial stage of their growth, and should be explored deeply to bring out a correlation between the technology and research. On the other hand, we should find a way to overcome the challenges, such as difficulty in modelling, collection reliable raw data and computational cost. The growing arena of computation and technology can be well utilized for the effective prediction of stable and productive photocatalyst materials. Hydrogen production and chemical fuels using water is one of the growing research areas in the latest decade (Fig. 32).

The photocatalytic method is probably the most economical means of hydrogen production compared to other methods, such as electro-catalysis, gasification, and pyrolysis. The photocatalytic method of producing hydrogen is gaining great attention and is widely obtained by the splitting of water. The same method can be used for water production using biomass as the substrate. This is an effective step to convert waste into valuable fuels. This can be carried out *via* the photo-reforming of sucrose, glucose, starch, and other biomass derivatives. H_2_ generation from renewable biomass presents an appealing possibility to fully realize the economic advantages of H_2_ as a fuel. The photo-reforming of biomass substrates has proven thermodynamic and kinetic advantages over the water splitting photoreaction. Still, this method has not been fully explored by the research community. Different photocatalysts have been developed and improved for higher efficiency and stability. OIH materials involve a photocatalyst based on carbon nitrate, graphitic oxide, and quantum dots, which have been discussed in this review. Still, metal–organic frameworks and covalent organic frameworks are not efficiently used for the hydrogen production from biomass and its substrates. However, the production of useable organics has been already successfully carried out by MOF functional materials. This showcases the wide range of opportunities available for the photocatalytic hydrogen production using OIH materials using biomass and its substrates. Apart from the challenges, biomass photoreforming can be considered as an emerging technology that can influence the production of green hydrogen and sustainable method for valuable chemical production. The future work shall focus on designing an efficient, selective and stable hybrid material with appropriate band gap for PR of biomass under sunlight, which is still under development. It is also important to use earth-abundant elements as a cocatalyst for better efficiency. The overall objective is to find an efficient, eco-friendly photocatalyst with lower driving force that produces H_2_ on a large scale using raw biomass materials. The ultimate aim is to use photoreforming to produce more valuable chemicals by the use of lower energy radiations, which is not suitable for water splitting reactions. This can be an efficient pathway to use the photoreforming mechanism for commercial and economical aspects. Although it is a long journey, we can expect future works to be based on these ideas and gaps to prepare suitable photocatalytic OIH materials for different applications. Photocatalysis provides a potential pathway to substitute the energy-consuming method of thermal reforming of biomass to produce hydrogen from solar energy. Hydrogen is a renewable, energy-efficient source of energy, and it has all sorts of possibilities to the future of green fuels. A strategy to produce hydrogen in a sustainable, efficient and pollution-free method should be developed, and photoreforming of biomass using solar energy can be considered as a possible solution.

## Author contributions

Ashil Augustin: investigation, writing – draft, review, Chitiphon Chuaicham: writing – draft, Mariyappan Shanmugam: writing – draft, Vellaichamy Balakumar: writing – draft, Saravanan Rajendran: writing – draft, Tuan K. A. Hoang: writing – draft, Keiko Sasaki: writing – draft, Karthikeyan Sekar: review, writing, and editing.

## Conflicts of interest

There are no conflicts to declare.

## Supplementary Material
